# Heat stress transcripts, differential expression, and profiling of heat stress tolerant gene *TaHsp90* in Indian wheat (*Triticum aestivum* L.) cv C306

**DOI:** 10.1371/journal.pone.0198293

**Published:** 2018-06-25

**Authors:** Harinder Vishwakarma, Alim Junaid, Jayanand Manjhi, Gyanendra Pratap Singh, Kishor Gaikwad, Jasdeep Chatrath Padaria

**Affiliations:** 1 National Research Centre on Plant Biotechnology, Pusa campus, New Delhi, India; 2 Shobhit University, Meerut, Uttar Pradesh, India; 3 Indian Institute of Wheat and Barley Research, Karnal, India; Université de Genève, SWITZERLAND

## Abstract

To generate a genetic resource of heat stress responsive genes/ESTs, suppression subtractive hybridization (SSH) library was constructed in a heat and drought stress tolerant Indian bread wheat cultivar C306. Ninety three days old plants during grain filling stage were subjected to heat stress at an elevated temperature of 37°C and 42°C for different time intervals (30 min, 1h, 2h, 4h, and 6h). Two subtractive cDNA libraries were prepared with RNA isolated from leaf samples at 37°C and 42°C heat stress. The ESTs obtained were reconfirmed by reverse northern dot blot hybridization. A total of 175 contigs and 403 singlets were obtained from 1728 ESTs by gene ontology analysis. Differential expression under heat stress was validated for a few selected genes (10) by qRT-PCR. A transcript showing homology to *Hsp90* was observed to be upregulated (7.6 fold) under heat stress in cv. C306. CDS of *TaHsp90* (Accession no. MF383197) was isolated from cv. C306 and characterized. Heterologous expression of *TaHsp90* was validated in *E*. *coli* BL21 and confirmed by protein gel blot and MALDI-TOF analysis. Computational based analysis was carried out to understand the molecular functioning of TaHsp90. The heat stress responsive SSH library developed led to identification of a number of heat responsive genes/ESTs, which can be utilized for unravelling the heat tolerance mechanism in wheat. Gene *TaHsp90* isolated and characterized in the present study can be utilized for developing heat tolerant transgenic crops.

## Introduction

Wheat (*Triticum aestivum* L.) ranks as the third largest food crop worldwide in terms of production. It provides an excellent source of many essential nutrients particularly vitamins, starch, minerals for 30% of the total world’s population [1]. According to climatic models, it is predicted that global temperature will continue to rise in coming years which will adversely affect agricultural production [2, 3, 4]. In India, Eastern Gangetic plains, Central and Peninsular parts of India are the major wheat growing areas and these are expected to be severely affected by heat stress [5]. Wheat is highly sensitive to high temperature stress especially at the post anthesis stage (i.e. grain filling) and temperature above the threshold at this stage leads to a drastically reduced yield [6]. Under conditions of high temperature stress, many metabolic and cellular changes take place in plants but the precise mechanism of high temperature stress tolerance is still unclear. Bread wheat cv. C306, a selection from cross between (Regent X Ch23) X C591 (P19 X C28), is a heat and drought tolerant genotype which is usually grown under rainfed conditions [7, 8]. Limited studies have been carried out to identify the genes associated with heat stress tolerance in cv. C306 [9, 10]. However, wheat cv. C306 is extensively used in breeding program for development of drought tolerant wheat as well as in the identification of QTL (Quantitative Trait Loci) linked to drought stress tolerance [8]. Bread wheat cv. C306 is a good genomic resource for understanding the molecular basis of heat stress tolerance in wheat. It can also be explored to harness heat stress responsive genes which can be utilized for developing heat stress tolerant wheat using breeding or transgenic approaches. Suppression Subtractive Hybridization (SSH) technique is an effective molecular biology tool for identifying differentially expressed genes through subtracted cDNA libraries. It is a low cost alternative to RNA sequencing methods for identifying differentially expressed, novel and/or uncharacterized genes/ESTs, even in organisms where sequence information available is only limited. Though SSH suffers from generation of low data resulting in poor genome coverage, yet it still continues to be one of the most popular technique for generating differentially expressed ESTs in any organism [11, 12, 13].

Exposure to high temperature activates cellular mechanisms responsible for heat stress, in response to which expression of a number of networking genes like Heat shock proteins (Hsps), molecular chaperones and other genes for interlinked metabolic enzymes are triggered. Chaperones like Hsp60/Hsp10 and Dnaj/Dnak/NEF (Nucleotide Exchange Factor) convert proteins misfolded due to stress to their active conformations whereas Caseinolytic proteases (Clp) play a major role in clearance of protein aggregates. Maize and rice mutants having disrupted ClpB/Hsp100 gene were observed to be susceptible to high temperature stress [14]. Hsp90 and Hsp70 are chaperones that regulate heat stress gene expression by interacting with Hsfs (Heat stress specific transcription factors) [15]. Hsp90 works in coordination with another important co-chaperone called as SGT1 (Suppressor of G2 Allele) by regulating auxin response. The interaction between Hsp90 and SGT1 plays a critical role in abiotic and biotic stress responses [16, 17, 18]. Hsp90 mutants in *Arabidopsis* were observed to have an abnormal embryo development and seed formation which confirms the crucial role of Hsp90 proteins in embryogenesis. [19]. HSP90 is a highly conserved molecular chaperone primarily involved in protein folding, activation and signal transduction. During initial investigations, these set of proteins were found to be upregulated in cells under stressed conditions, giving the term heat shock protein of 90 kDa. Hsp90 is mainly composed of three conserved domains, N-terminal ATPase domain (ND), middle domain (MD) and C-terminal dimerization domain (CD) [20]. This study aimed at identifying differentially expressed genes under heat stress conditions in wheat cv. C306 at post anthesis stage by carrying out a detailed transcriptome analysis through SSH technique. Reverse northern dot blot was used to pick up most appropriate heat stress related transcripts prior to sequencing. Few potential heat stress responsive transcripts were validated by qRT-PCR analysis. One of the highly expressed transcript identified in the study, *TaHsp90* (Accession no. MF383197) was isolated and characterized for *In-silico* and computational based analysis to understand its molecular functioning and interaction with other molecules. The gene *TaHsp90* was cloned in prokaryotic expression vector pET28a (+) and was functionally validated in *E*. *coli*. The *E*. *coli* cells having recombinant plasmid (pET28-*TaHsp90*) was able to provide heat stress tolerance to *E*. *coli* cells under high temperature conditions. This gene can be validated for heat stress tolerance in a model plant system with an aim to develop transgenic crops for climate resilient agriculture. The heat responsive EST data generated in wheat cv. C306 would open a new avenue to understand molecular basis of stress tolerance in crop plants be and the identified unique transcripts related to heat stress response can be deployed for development of heat stress tolerant transgenics.

## Materials and methods

### Plant materials, growth conditions and stress treatment

The seeds of Indian bread wheat cv. C306 (heat and drought tolerant) and HD2967 (heat susceptible) used in the study were obtained from the Division of Genetics, IARI, New Delhi, India. Pre-vernalized seeds of both the genotypes were transferred to seven inches pots containing soilrite and grown under green house conditions. Normale growth conditions were maintained with temperature of 24±2°C, light intensity of 350 μmol/m^2^/s with photoperiod for 16/8 h and 60% humidity. Leaf samples were collected at post-anthesis stage (Feekes scale-10.53) after giving heat stress (HS) at 37°C & 42°C for different time intervals i.e. 30min, 1h, 2h, 4h & 6h. 1 g of leaf tissue was collected at different time intervals of stress from three biological replicates. Heat stress treatment was carried out in an incubator chamber by incremental of 1°C temperature per 10 min until it reached the desired HS temperature [21]. After heat stress treatment the controlled conditions were brought down in same manner. The plants grown without HS treatment (24±2°C) were used as control. Leaf samples were harvested following heat stress and immediately frozen in liquid nitrogen and stored at -80°C for further molecular biology experimentation.

### Construction of cDNA library using SSH

Total RNA from wheat cv. C306 was isolated from leaf samples using Spectrum Plant RNA isolation kit (Sigma, USA). The RNA samples were pooled for SSH library construction. The quantity and quality of RNA was checked on Bioanalyzer 2100 (Agilent, USA) using RNA Nano-chip (Agilent, USA) as per the manufacturer’s instructions. A forward SSH library was constructed using Clontech PCR Select^™^ cDNA subtraction kit (Clontech, USA) as per manufacturer’s instructions. Differentially expressed cDNAs having different adaptors 1A and 2R at two ends were amplified by PCR, a secondary PCR was done to further reduce any background PCR product and enrich for differentially expressed sequences. After purification with QIA PCR purification kit (Qiagen, USA), the secondary PCR products obtained were ligated to pGEM-T easy vector (Promega, USA) and transformed to Electromax DH10β *E*. *coli* cells (Invitrogen, USA) by electric shock method using electroporator gene pulser (BIORAD, USA) at 1.8 kVa for 6 ms and plated on LA (Luria-Bertani Agar) plate supplemented with 100 μg/ml ampilcillin, 1 mM IPTG (Isopropyl β-D thiogalactopyranoside) & 40 μg/ml X-gal (5-bromo-4-chloro-3-indolyl-β-D-galactopyranoside). The plates were incubated at 37°C for 16h and then at 4°C for 2h so as to clearly visualize the blue-white colonies. Only white colonies were picked up and checked with T7 & SP6 primer pairs for the cDNA inserts. Clones having insert less than 200 bp were not taken further.

### Reverse northern blot analysis

Reverse northern dot blot analysis was performed with labelled total cDNA of wheat cv. C306 (control and HS treated) using DIG high prime DNA labelling and detection starter kit (Roche, Germany). 2 μl of each colony PCR product (10 ng) was spotted directly on a sheet of positively charged nylon membrane (Millipore, USA) in a 96 well plate manner using Bio dot filtration apparatus (Biorad, USA) attached with a vaccum pump. After transfer, the membrane was cross linked at 1.2 J/cm^2^ for 2 min to fix the nucleic acid to the membrane. Pre-hybridization, using Hyb granules as pre-hybridization solution, was done at 50°C (calculated as per manufacturer’s instructions) for 1 h followed by hybridization in a hybridizer (HM-4000, India) at the same temperature for 16 h. After hybridization, the sheet was washed twice in 2X SSC containing 0.1% SDS at room temperature (25°C) for 5 min followed by washing twice in 0.5X SSC containing 0.1% SDS at 65°C for 15 min. Blocking of membrane for 30 min followed by incubating with Anti-DIG antibody solution having alkaline phosphatase enzyme, the membrane was subjected to immunological detection using NBT/BCIP as substrate solution supplied in DIG DNA labelling and detection kit, according to manufacturer’s instructions. The differential expression of each spot was recorded. The positive clones were selected for sequencing (ABI Prism 310, Applied Biosystems, USA). The library was stored at -80°C in 25% filter sterilized glycerol in 96 well plate for future use.

### DNA sequencing and annotation

A total of 2000 clones, having insert size of 200 bp and above (1000 clones from 37°C HS forward library and 1000 clones from 42°C HS forward library) were randomly selected for Sanger’s sequencing after PCR confirmation and *Eco*RI restriction digestion analysis. All the raw data were first screened for removal of vector and adaptor sequences using VecScreen software tool (http://www.ncbi.nlm.nih.gov/tools/vecscreen/). Further the low quality and short sequences (<100 bp) were not taken into consideration. Homology search was conducted using BLASTN and BLASTX algorithm search tool (http://www.ncbi.nlm.gov/BLAST) with a cut-off value of 1.0 E-3. CAP3 program was used for assembling the sequence reads with default parameters [22]. BLAST2GO (https://www.blast2go.com/blast2go-pro) software tool was used for functional annotation of contigs and ESTs (parameters: nr database, HSP cutoff length 33, report 20 hits, E value 1.0E-3, annotation cutoff 55, HSP hit avg cutoff 20). The ESTs were further categorized on the basis of biological function, molecular function & cellular component. KEGG (Kyoto Encyclopedia of Genes and Genomes) pathway and enzyme code distribution related to HS were also analyzed using BLAST2GO. TMHMM transmembrane motifs were also detected by BLAST2GO software using default parameters settings. Subsequently GO terms of annotated ESTs were retrieved and used for singular enrichment analysis (SEA) using AgriGO ((http://bioinfo.cau.edu.cn/agriGO/). To perform SEA, wheat Affymetrix genome array database was used as background reference. GO catergories having *p*-value ≤ 0.05 were used further for interactive map analysis using REViGO software tool [23]

### Real-time PCR analysis

To validate the expression under HS of the deduced genes in forward SSH libraries, qRT-PCR analysis was carried out. Total RNA was isolated from leaf samples of each HS treatment from wheat cv. C306 and cv. HD2967 using QIA Plant total RNA kit (Qiagen, USA). RNA was also isolated from control (no HS) samples. On column DNase treatment (Sigma-Aldrich, USA) was given to remove any DNA contamination. The eluted RNA was quantified on Nano LC (Thermofisher Scientific, USA). cDNA was synthesized from the RNA isolated for each timeline of stress using Superscript III^RT^ first strand cDNA synthesis system (Invitrogen, USA). Primers were designed for qRT-PCR analysis of the ten selected deduced ESTs {*Hsp90* (heat shock protein 90), hypothetical *Dnaj*, *Hsp-Sti* (heat shock protein-stress inducible), hypothetical *Hsp*, ClpB1/Hsp100, Peptidyl prolyl cis-trans isomerase (FKBP), Peptidyl prolyl isomerase (PPIase), Glyceraldehyde-3 phosphate dehydrogenase (*GAPDH*), ATP cleavage protease (AAA ATPase), Photosynthetic Reaction Centre Protein (PSBR)} using Primer quest software tool (http://www.idt.com) (Table 1). Each PCR reaction (10 μl) consisted of 5 μl Lightcycler 480 SYBR green Master mix (Roche, Germany), 4 μl diluted cDNA (100 ng), and 0.5 μM forward and reverse primers. Amplification was carried out at 95°C for 3 min, followed by 40 cycles (95°C for 10s, 60°C for 10 s, 72°C for 10 s). *β-actin* gene (accession no. AB181991.1) was used as internal control, after assessing its stability to be used as reference gene [24]. Each reaction was performed in triplicates and relative fold change values between experimental and calibrator sample was calculated by 2^-ΔΔCt^ method [25]. The primer-template specificity was monitored by melting curve analysis. The primers used in the study are shown in Table 1.

**Table 1:** List of primers used in the study.

	Name of the primer	5′-3′ sequence	Amplicon obtained (bp)
1	rTaHsp90 fl Nde F	GGGTTTCATATGATGGCTTCGGAGACCGAGACC	2103
2	rTaHsp90 fl Xho R	GGCCTCGAGCTAGTCGACCTCCTCCATCTT	
3	qactin F	GAAGCTGCAGGTATCCATGAGACC	125
4	qactin R	AGGCAGTGATCTCCTTGCTCATC	
5	q229hsp90 F	CTTCTCAAGGAAGGGAGAGTTC	97
6	q229hsp90 R	GGACTATGTGACGAGGATGAAG	
7	qhspsti F	TGCTCGGATCATGCTTTAGG	109
8	qhspsti R	CCTACATTCTCCAAGGGATACAC	
9	qClpB1 F	GTTCTCACGCCTCAGTCATT	140
10	qClpB1 R	GATGTAAGCTCCGTGTCGATAG	
11	qDnaj F	GCCAGGAGACCTTTATGTCTTTA	119
12	qDnaj R	CAGTTGTCCCAAGAATTGCATC	
13	qPPI F	CGTAGCGACTGGACCAAATAA	107
14	qPPI R	TCAAGGCAGCTCTAGGAAATG	
15	qPPCTI F	TAACGCAGGCCCAAACA	112
16	qPPCTI R	CATAGATTCTTCGTCCGCTACTT	
17	qG3PD F	CTCACTGTTAGAACCGAGAAGG	125
18	qG3PD R	AAGTCGGTGGAGACCAAATC	
19	qATP clp F	TAACCAGAGCAATGTTCGAGAG	132
20	qATP clp R	CTTCTCCAACCAGACCCAATAG	
21	qPRCP F	ACCTAAAGCAGTGAACCAGATT	124
22	qPRCP R	TGTGGCTGCTCATGGTTATT	
23	qhyp hsp F	GAGTGAAGGAATGAGGGATGAA	103
24	q hyp hsp R	CGCTGTTACTTAAATGACCCATAC	

Restriction sites are underlined

### *In-silico* analysis of TaHsp90

Different protein parameters were included in *in-silico* analysis: amino acid composition, total number of positively and negatively charged residues, atomic composition, instability index, hydropathicity index, and others were measured using Protparam (http://web.expasy.org/cgi-bin/protparam) software. Phylogenetic tree was constructed in MEGA7 software tool using Neighbour-joining method for studying evolutionary relationship with *Hsp90* of other plant species based on the nucleotide and deduced amino acid sequences. The boot strap value was set as 1500 and the evolutionary distance was calculated using *p*-distance method. Multiple alignment of TaHsp90 with Hsp90 of other related species was carried out using Clustal Omega (https://www.ebi.ac.uk/Tools/msa/clustalo/).

#### Subcellular location, phosphorylation site and kinase specific prediction

The subcelluar location of TaHsp90 was predicted by using software CELLO v.2.5 (http://cello.life.nctu.edu). To predict the phosphorylation profile analysis and kinase specific prediction Netphos 3.1a server was used (http://cbs.dtu.dk/services/Netphos/). Transmembrane helix region was predicted by using TMHMM server (http://www.cbs.dtu.dk/services/TMHMM/). NetNGly 1.0 server and YinOYang 1.2 server were used to find out the N-linked and O-linked glycosylation respectively in TaHsp90.

#### Secondary structure prediction and modelling

The α-helices, β-strand and coils were predicted using PSIPRED software tool (http://bioinf.cs.ucl.ac.uk/psipred/). Amino acid percentage composition was also calculated using the same software tool. The disordness in the secondary structure of protein was predicted using Phyre^2^ software tool (http://www.sbg.bio.ic.ac.uk/phyre2). For normalized QMEAN4 score and 3D protein structure prediction Phyre^2^ (http://www.sbg.bio.ic.ac.uk/phyre2) software tool was used. Further the generated model was docked to a ligand using 3DLigand Site prediction server (http://www.sbg.bio.ic.ac.uk/3dligandsite) to find where would a ligand binds to TaHsp90. For active site prediction of TaHsp90 ligand 3D site online tool was used.

#### Ramachandran plot and TaHsp90 predicted expression analysis in wheat

The conformations of the obtained 3D structure was inspected by Ramachandran plot and the structure quality was calculated in terms of amino acid residues percentage in favourable regions [26, 27]. The Ramachandran plot was verified by using Verify 3D and ERRAT software tool. For digital expression based studies, Genevestigator software tool (https://genevestigator.com/) was used. The probeset encoding Hsp90 from *Triticum aestivum*, *Oryza sativa*, *Zea mays* and *Hordeum vulgare* were retrieved from the publicly available Affymetrix database using and temporal and spatial levels of expression of *TaHsp90* was assessed at 10 different developmental stages (germination, seedling, tillering, stem elongation or jointing, booting, heading, flowering or anthesis, milk, dough, ripening) and in 26 important anatomical parts (seedling, coleoptile, leaf shoot apex, mesocotyl, crown, root, inflorescence, spike, spikelet, floret, stamen, anther, pistil, glume, caryopsis, embryo, endosperm, shoot, leaf, sheath, flag leaf, blade (lamina), crown, rhizome, roots) using the identified probeset Ta.9140.2S1_at. Further, *in silico* studies were carried to find out the most similar related genes that matched with TaHsp90 usingthe probeset Ta.9140.2S1_at, and the relative expression of these genes was analyzed in 26 anatomical parts of wheat (as mentioned above). The total number of target genes were set at 100, however Pearson’s correlation coefficient of TaHsp90 was analyzed with the most correlated top 25 genesby setting the correlation coefficient cut off at 0.796. Digital based expression studies of *TaHsp90* was also compared at different developmental stages with *Hsp90* of rice, maize and barley. A protein to protein interaction of TaHsp90 was studied by string based interaction (https://string-db.org). The string analysis was performed based on *Hordeum vulgare* which is most closely related to wheat (Family Triticaceae), as the *Triticum aestivum* was not listed among the organism list in string software tool.

### Construct preparation and transformation in *E*. *coli* BL21 cells

To obtain full length *Hsp90* of *T*. *aestivum* cv. C306 (Accession no. MF383197), gene specific primers were designed. The ORF was amplified from total cDNA of wheat cv. C306 with primer having *Nde*I & *Xho*I site respectively. PCR was carried out using proof reading Advantage 2 DNA polymerase (Clontech, USA). A three step PCR was performed (94°C for 2 min, then 29 cycles of 94°C for 30 s, 58°C for 30 s, 72°C for 2 min and final extension at 72°C for 7 min [28]. The product was cloned under the control of T7 promoter in pET28a (+) vector digested with *Nde*I & *Xho*I restriction enzymes. The orientation of insert in resulting recombinant construct pET28-*TaHsp90* was verified by PCR using T7 promoter primer and gene specific reverse primer. Further construct was confirmed by restriction digestion with *Nde*I and *Xho*I enzymes. The resultant construct was transformed to *E*. *coli* BL21 (DE3) cells (Invitrogen, USA) using heat shock method. Clones obtained were verified by colony PCR. The cells (positive clones) were grown in 5 ml LB supplemented with 30 μg/ml kanamycin at 37°C, 200 rpm and then induced with 1mM IPTG when O.D was 0.5 at A_600_. The cells were collected after 6 h of induction and total protein was isolated [29].

### Protein determination, SDS-PAGE and protein gel blot and heat stress tolerance assay

Total protein was estimated by Bradford method (Biorad, USA) using BSA (Bovine Serum Albumin) as standard. 20 μg of protein sample was pre treated with SDS lamelli buffer (200 mM Tris-HCl pH 6.8, 10% SDS, 10% glycerol, 0.25% Bromophenol blue having 5% β-mercaptoethanol) and incubated at 100°C for 10 min. The running buffer was composed of 0.3% Tris base, 1.3% Glycine, 0.1% SDS. Two replica gels having 12% resolving and 5% stacking polyacrylamide gel were prepared and the protein was electrophoresed at room temperature [30]. Pre-stained protein marker (Fermentas, USA) was used as a molecular weight marker.

When the the bromophenol blue (tracking dye) reached bottom of the gel, the gel was stained for 20 min with Coomassie stain (Coomasssie brilliant blue (CBB) R250 & G250 (Biorad, USA):0.125% each, 40% methanol, 10% acetic acid in water) followed by destaining by keeping in a destaining solution (40% methanol, 10% acetic acid in water) for overnight. The replica gel was transferred to PVDF membrane (Biorad, USA) using transfer module cassette (Biorad, USA) in transfer buffer (0.3% Tris, 1.3% Glycine) at 40 V for 1h. Presence of prestatined marker on the membrane confirmed the transfer process. The blot was then blocked with 3% BSA (Bovine Serum albumin) (Himedia, India) in TBST buffer (50 mM Tris-HCl pH-7, 0.9% NaCl, 0.02% SDS, 0.1% Tween 20) and incubated on shaker for 30 min. Anti-His-tag monoclonal antibody (Abm, Canada) diluted in blocking solution at 1:4000 dilution and incubated on shaker for 1h, then the blot was washed with TBST buffer for 3 times (10 min each). Anti-IgG (anti-mouse) antibody having alkaline phosphatase was used as secondary antibody (1:4000 dilution) and incubated on shaker for 1h. 3 washes with TBST buffer were given for 10 min each. The membrane was developed with NBT/BCIP substrate solution (Sigma, USA) by incubating the membrane in dark for 2–3 min, the reaction was stopped by adding autoclaved double distilled water. The recombinant *E*. *coli* BL21 cells carrying construct pET28-*TaHsp90* and pET28 vector alone were grown in liquid medium to log phase and induced with IPTG (1mM). The cultures were subjected to heat stress at 45°C for 6h. The culture growing at 37°C were used as control. Thereafter the cultures were serially diluted (10^−3^, 10^−4^ and 100 μl was spread on LA plates supplemented with 30 mg/l kanamycin. The plates were incubated at 37°C for overnight, number of colonies were counted and CFU (colony forming unit) was calculated as per given formula:

CFU/ml=no.ofcolonies*dilutionfactor/volumeofcultureplate

### Protein purification by Immobilised metal affinity chromatography (IMAC), sample prep for MALDI

50 μg of soluble protein was taken having 20 mM imidazole, loaded on His-trap column (GE healthcare, UK) pre-equilibrated with 20 mM imidazole buffer (200 μl, 3X times). The column was then washed with 20 mM imidazole buffer (200μl, 3X times) further eluted with 40 μl of 500 mM imidazole buffer. Different fractions like flow through, washes and elutions were loaded on SDS-PAGE. To facilitate identification of purified protein, single band was excised from CBB stained gel and washed twice with milli-Q water for 15 min. The sliced gel was chopped into fine pieces, destained and then dehydrated using acetonotrile until gel pieces turned opaque and shrunk in size. The solution was dried in speed vac and then treated with DTT (Dithiothreitol) and iodoacetamide, supernatant was collected, gel pieces were incubated with ammonium bicarbonate (NH_4_HCO_3_). Supernatant was again collected, gel pieces were dehydrated with acetonitrile followed by speedvac. In gel digestion was carried out using proteomics grade Trypsin enzyme (Promega, Madison USA), 12.5 ng/μl of enzyme in a volume of 10 μl. The reaction was incubated at 37°C overnight then the supernatant which is now having the digested peptides was extracted thrice in 50% acetonitrile having 0.1% trifluoroacetic acid. The mixture was vaccum dried in speedvac followed by mixing with HCCA (α-cyano-4-hydroxycinnamic acid) and then spotted onto MALDI plate. Mass spectra (m/z) was acquired using MALDI TOF/TOF ULTRAFLEX III instrument (Bruker, Germany), which was analyzed with FLEX ANALYSIS SOFTWARE for PMF (Peptide Mass Fingerprinting), the data obtained was searched in the protein database for identification using search engine Matrix science (http://www.matrixscience.com. The following search parameters were taken: peptide mass tolerance of ±100 rpm, one incomplete cleavage, oxidation of methionine as variable modification, modification of carabamidomethyl was kept fixed, protein mass values unrestricted, monoisotopic mass value and +1 peptide charged state [29].

## Results

### RNA isolation, cDNA synthesis and reverse northern dot blot

**Fig 1:**
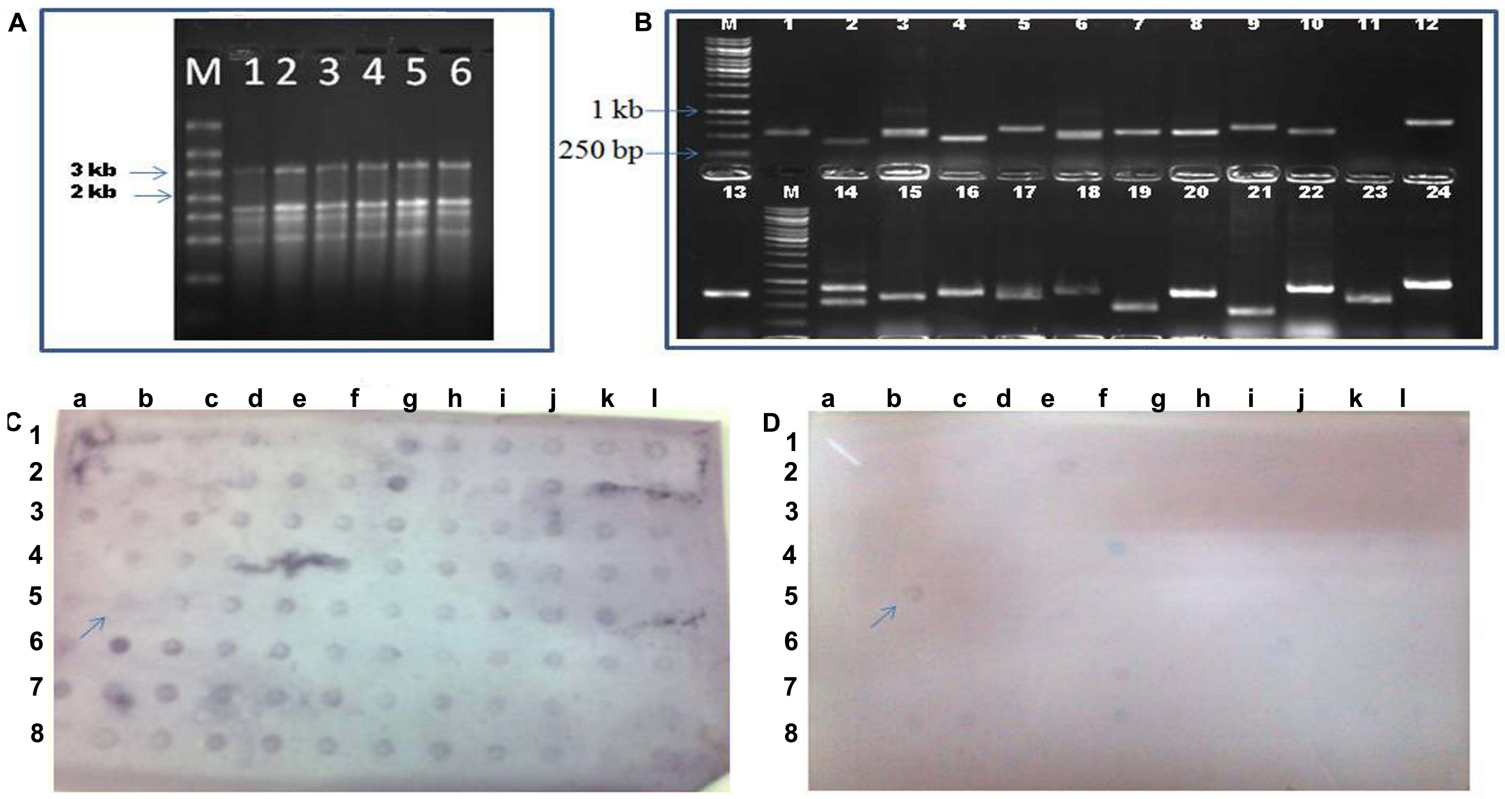
Construction of SSH library and reverse northern dot blot. (A) Total RNA isolated from control and treated plants. Lane M-RNA ladder, lane 1, 2 Wheat cv. C306 control plant RNA, lane 3, 4 wheat cv. C306 37°C- stressed plant RNA, lane 5, 6- wheat cv. C306- 42°C stressed plant RNA. (B) PCR of the obtained clones after library preparation using T7/SP6 primer pairs, M is the 1 kb DNA marker. (C) Reverse northern dot-blot, Probe prepared from heat stressed plants-total cDNA. (D)-Probe prepared from control plants-total cDNA, the arrow (→) mark indicates the positive control (PCR product of *β-actin*, a housekeeping gene).

Forward subtraction library was prepared from total RNA isolated from leaf tissue of wheat cv. C306 at post anthesis stage subjected to HS (37°C and 42°C) (Fig 1A). cDNA synthesis was observed as smear in the range of 100 bp to 1.5 kb on 1.5% TBE gel. PCR analysis of the 1000 clones picked from each subtracted (forward) library i.e. 37°C HS and 42°C HS showed inserts in the range from 100 bp to 1 kb (Fig 1B). A total of 950 clones from both libraries each (i.e. 1850 overall) which displayed at least 1.5 fold level of hybridization in dot blot analysis were selected for sequencing (Fig 1C and 1D). VecScreen analysis of the EST sequences obtained, generated 1728 good ESTs (848 ESTs from 37°C stressed library & 880 ESTs from 42°C stressed library) in the range of 124 to 700 bp size.

### ESTs assembly, functional annotation & qRT-PCR

**Fig 2:**
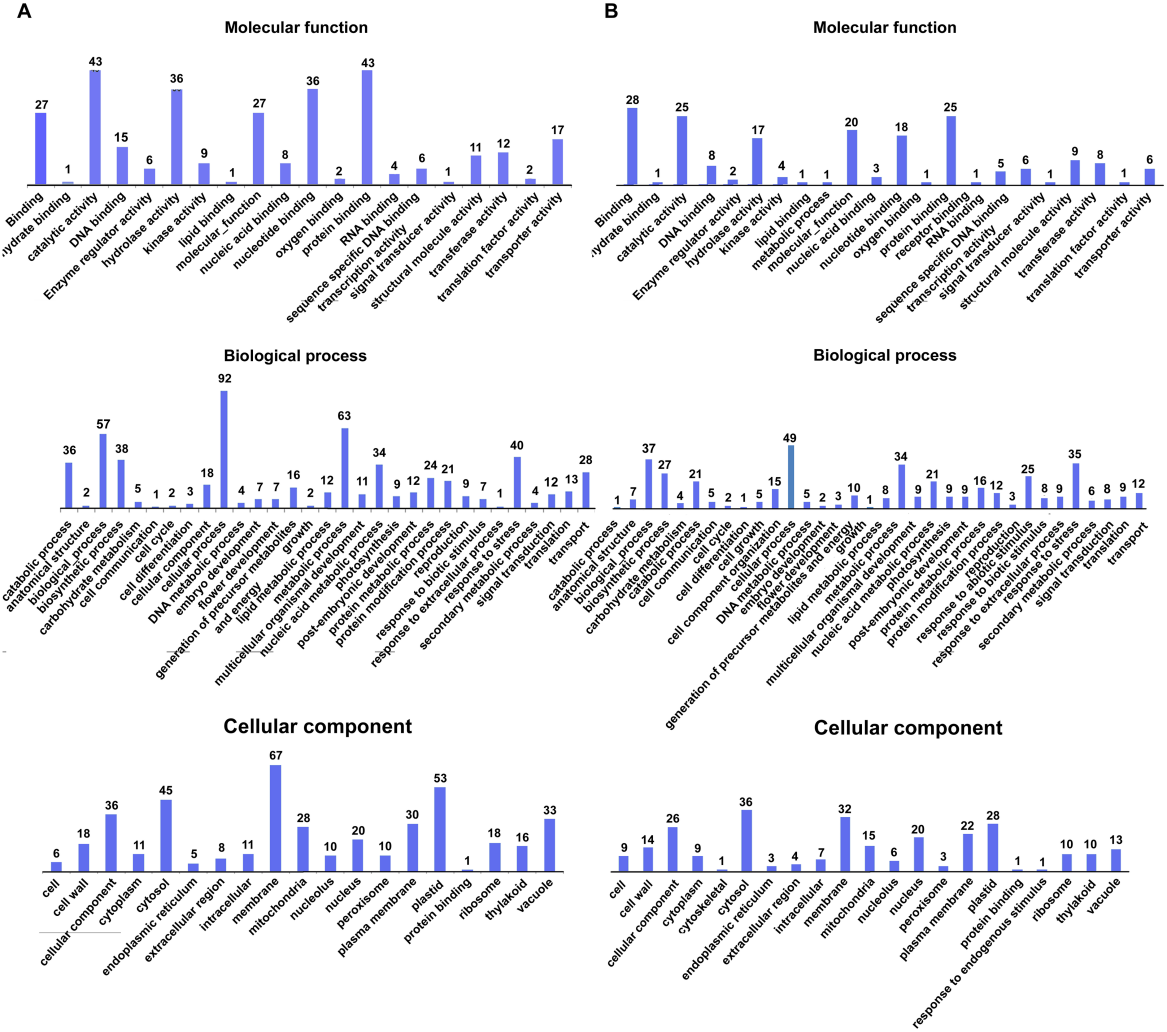
GO annotation analysis of assembled ESTs. Bar diagram showing distribution of assembled ESTs annotation to various molecular functions, biological processes and cellular component. (A) Wheat cv. C306 37°C HS forward library. (B) Wheat cv. C306 42°C HS forward library.

37°C HS forward library had 848 ESTs assembled to 115 contigs (5.2% did not match to the database) and 196 singlets (6.1% did not match) while 42°C HS forward library had 880 ESTs assembled to 60 contigs (10% did not match) and 207 singlets (20.77% did not match). All the ESTs have been submitted to EMBL-EBI database (Accession no. LT965080-LT965927: 37°C forward HS library; LT963784-LT964663: 42°C forward HS library). The Gene Ontology (GO) analysis was performed for unigenes sets obtained from 37°C & 42°C HS libraries separately. The average length of ESTs from 37°C and 42°C forward stress library were 359 bp and 517 bp respectively (S1 Fig). The ESTs classified into three main components i.e. molecular function, biological processes & cellular component (Fig 2A and 2B). In the molecular function category catalytic activity/protein binding & “binding’ ESTs were most prevalent in 37°C and 42°C stress library respectively. In the biological process category ESTs belonging to cellular processes were most prevalent in both category while in cellular component category membrane related & cytosol related ESTs were most prevalent in 37°C and 42°C HS library respectively. Under the biological process category we found the sequences related to protein folding (GO:0006457), response to stress (GO: 006950), translation process (GO: 0006412) with significant *p*-value (S1 Table). The Go annotations analysis in SSH libraries of 37 °C HS and 42°C HS showed that though the number of sequences in each prevalent category varied between both SSH libraries but the prevalent categories were observed to be same (Fig 2A and 2B).

Different types of 55 KEGG pathways were affected by the HS. The KEGG pathway analysis has shown that ESTs from subtracted library fall into the different categories as: glycolysis/gluconeogenesis pathway, TCA (Citrate) cycle, pentose phosphate pathway, galactose metabolism, ascorbate and aldarate metabolism, fatty acid metabolism, ketone bodies metabolism, steroid biosynthesis, oxidative phophorylation, purine metabolism, alanine, aspartate and glutamate metabolism, glycine, serine and threonine metabolism, cysteine and methionine metabolism, valine, leucine and isoleucine metabolism, geraniol degradation, lysine biosynthesis and degradation, tyrosine and phenylalanine metabolism, benzoate degradation, tryptophan metabolism, β-alanine metabolism, selenocompound metabolism, amino sugar and nucleotide sugar metabolism. The KEGG analysis revealed that important cellular pathways related to glutathione metabolism, starch and sucrose metabolism, metabolism of xenobiotics by cyt P450 were also among the ones that were affected by heat. It was deduced that ESTs from HS libraries having homology to enzymes belonging to enzymatic group oxidoreductases (18%) and transferases (7%) were most prevalent in the 37°C HS and 42°C HS libraries respectively (S2 Fig). BLAST2GO was used to generate the data/graph related to GO categories i.e. molecular function, biological processes and cellular component (S3 Fig). The GO terms involved in the KEGG pathway were summarized and shown as interactive map (S4 Fig).

**Fig 3:**
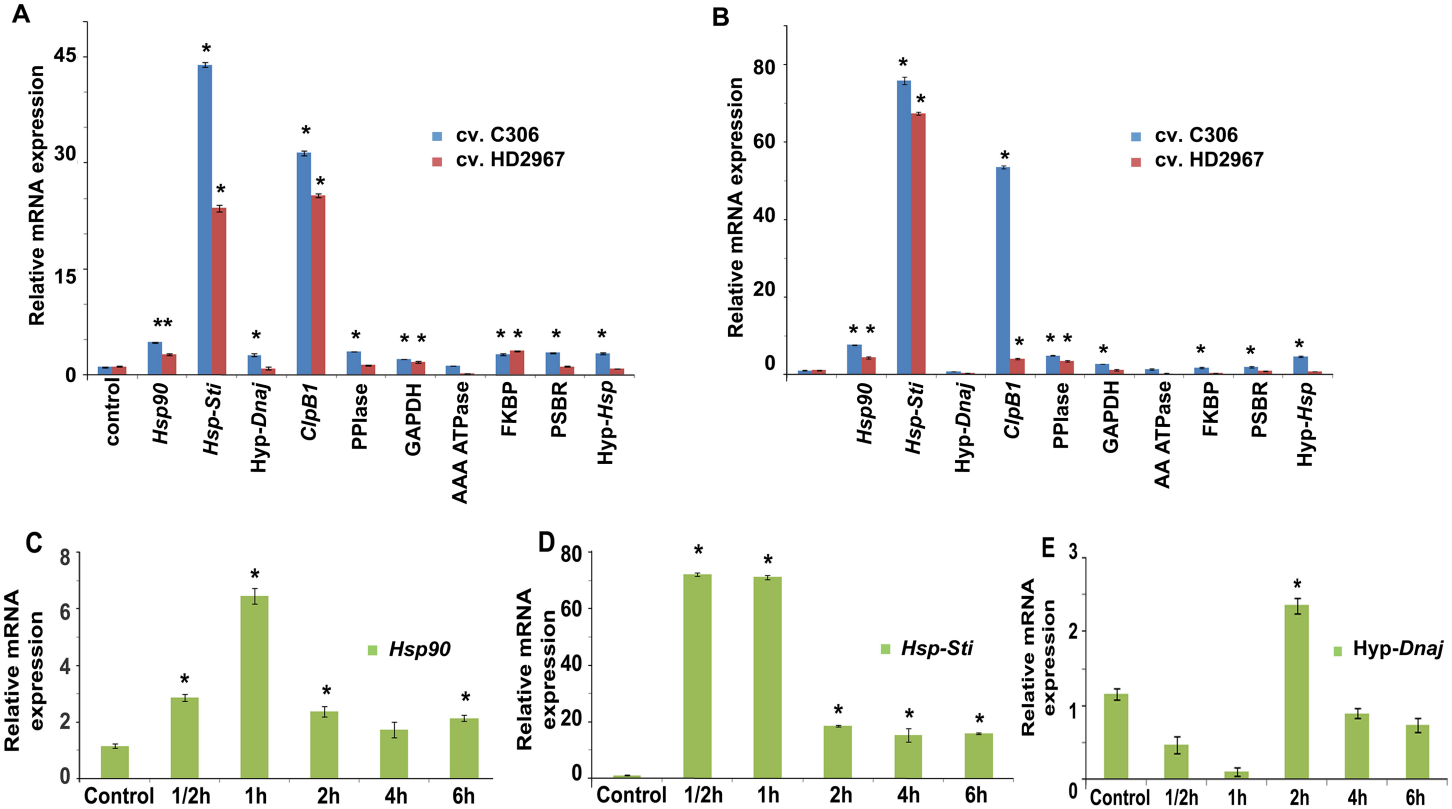
Gene expression analysis by qRT-PCR in contrasting wheat cultivars C306 (heat tolerant) and HD2967 (heat susceptible). (A) Fold change expression of Hsp90, *Hsp-Sti*, hypothetical *Dnaj*, ClpB1/Hsp100, PPIase, *GAPDH*, AAA ATPase, FKBP, PSBR, hypothetical *Hsp* at 37°C. (B) Fold change expression at 42°C. (C) Fold change expression of *Hsp90* in cv. C306. (D) Fold change expression of *Hsp-Sti* in wheat cv. C306. (E) Fold change expression of hyp *Dnaj* in wheat cv. C306. Statistical significance has been shown by asterisk (*) at p≤0.05 (n = 3).

qRT-PCR analysis of heat responsive candidate genes showed that *Hsp90*, *Hsp-Sti*, hypothetical *Dnaj*, *ClpB1*, *PPIase*, *GAPDH*, *PSBR*, hypothetical-*Hsp* were upregulated in response to high temperature stress (37°C and 42°C) in *T*. *aestivum* cv. C306 as compared to HD2967 (Fig 3A and 3B). These genes were further checked at different time intervals in wheat cv. C306 and it was observed that highest fold change gene expression of *Hsp90*, *Dnaj & Hsp-Sti* was at 30min, 1h & 2h respectively (Fig 3C–3E).

### *In-silico* and computational based analysis of TaHsp90

The TaHsp90 protein was found to have Glutamate (12.4%) as the highest and Cysteine (0.7%) as the lowest amino acid. The total number of negatively charged residues (Asp+Glu) were 140 while total number of positively charged residues (Arg+Lys) were 107, only which might be the reason for its acidic pI (4.97). The instability index and GRAVY (Grand average of hydropathicity) values were 35.35 & -0.614 which suggests its stable and hydrophilic nature. The stable nature of protein may be accounted for by a soluble protein formed after *TaHsp90* overexpression in *E*. *coli* BL21 cells. Theoretically, the molecular weight was predicted to be 80.425 kDa having an isoelectric point of 4.97, with atomic composition of C_3570_H_5686_N_928_O_1132_S_22_. Multiple sequence alignment of TaHsp90 with Hsp90 of other plant species and pairwise sequence comparison was done for nucleotides as well as deduced amino acids has been shown in Table 2 (S5 Fig).

**Table 2:** List of TaHsp90 genes retrieved from NCBI database for analyzing nucleotides and amino acid sequences.

Plant	Family	Accession no	Sequence length CDS (bp)	Deduced amino acid	% Sequence similarity Nucleotide	Amino acid
*Triticum aestivum*	Poaceae	GQ240776.1	2103	700	99	99
*Aegilops tauschii*	Poaceae	XM_020301926.1	2103	700	98	99
*Hordeum vulgare*	Poaceae	AK355136.1	2103	700	97	99
*Elymus sibiricus*	Poaceae	KM604224.1	2100	699	94	97
*Dactylis glomerata*	Poaceae	FJ968745.1	2100	699	93	95
*Brachypodium distachyon*	Poaceae	XM_003574679.3	2100	699	92	96
*Triticum turgidum*	Poaceae	GQ240799.1	2103	700	99	99
*Sorghum bicolour*	Poaceae	XM_002444759.2	2097	698	91	94
*Saccharum* hybrid cv. SP80-3280	Poaceae	JX992841.1	2097	698	91	93
*Oryza sativa japonica*	Poaceae	XM_015794192.1	2100	699	91	94
*Triticum urartu*	Poaceae	GQ240791.1	2103	700	98	94
*Oryza brachyantha*	Poaceae	XM_006659488.2	2100	699	90	94
*Pennisetum glaucum*	Poaceae	HM596079.1	2097	698	90	93
*Zea mays*	Poaceae	NM_001177009.1	2100	699	90	93
*Setaria italica*	Poaceae	XM_004973767.3	2097	698	90	92
*Secale cereale*	Poaceae	JQ685506.1	2103	700	89	94

**Fig 4:**
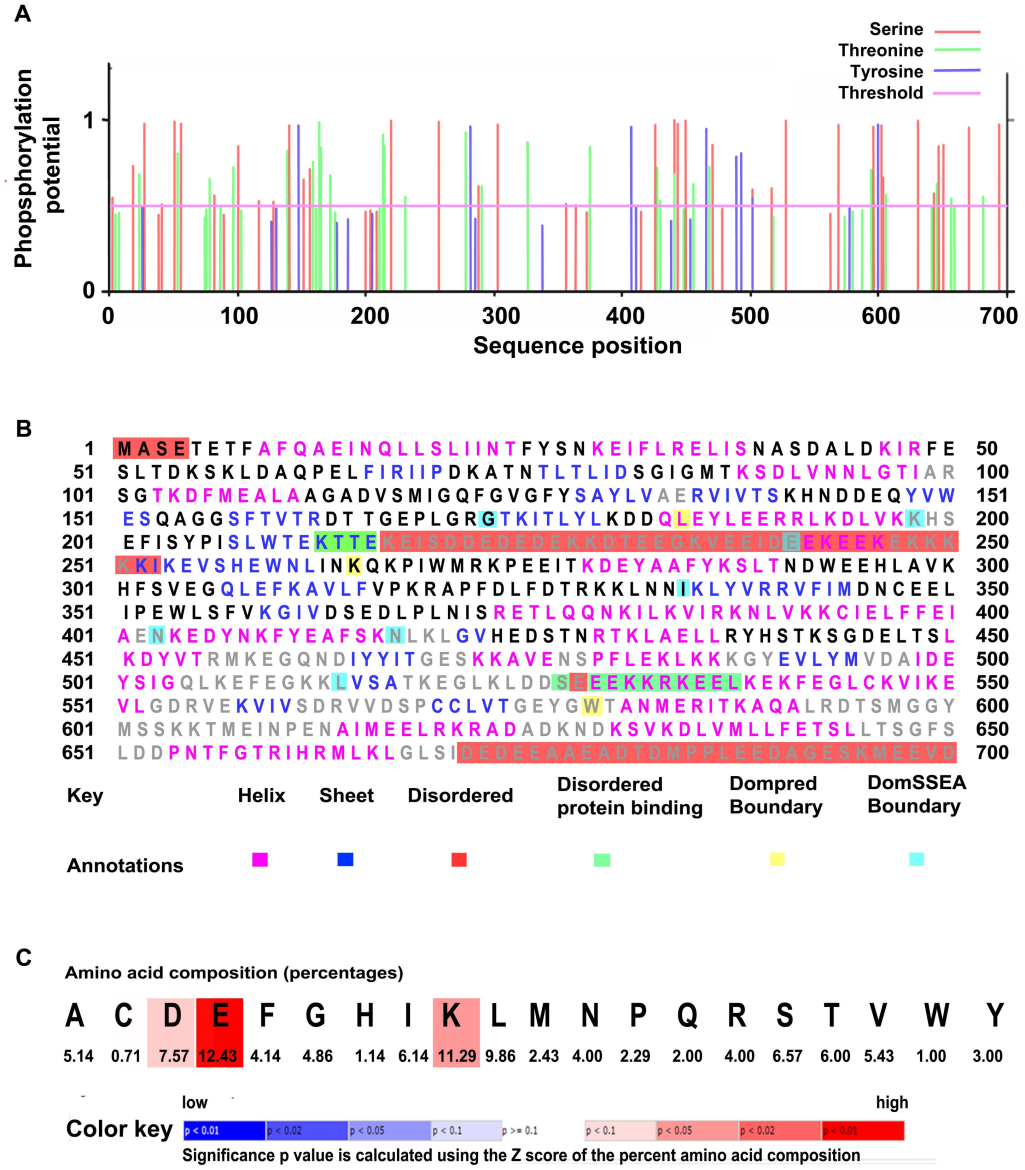
TaHsp90 phosphorylated sites and secondary structure prediction. (A) Phosphorylated sites prediction in TaHsp90 using Netphos 3.1a server. (B) Secondary structure map of TaHsp90 shows helices, sheets and disorderness. (C) Amino acid composition percentage along with the predicted significance p value. Feature predictions are color coded according to the sequence feature key shown below.

**Fig 5:**
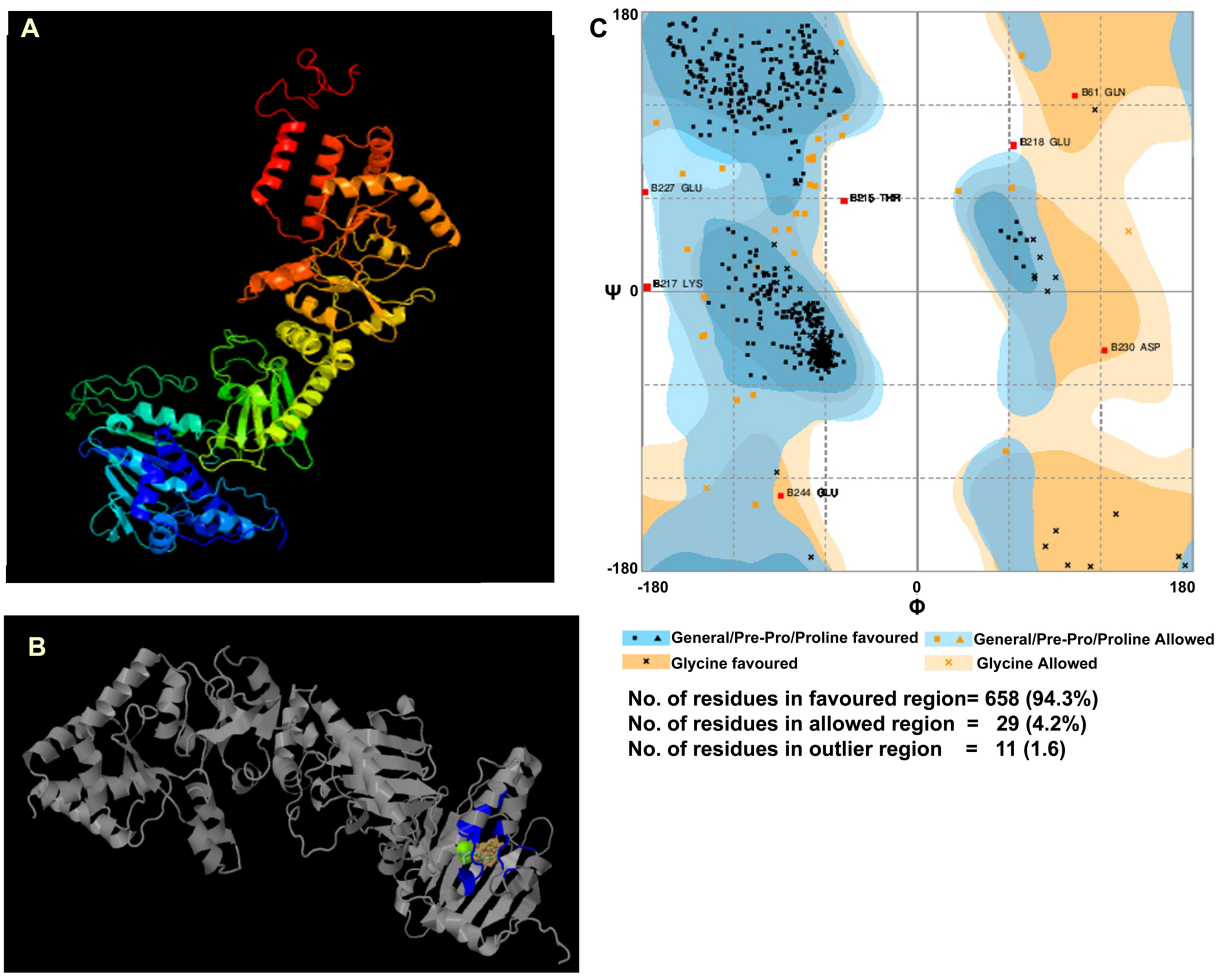
3D structure prediction of TaHsp90. (A) Modelling of TaHsp90 based on template-c5fwkA. (B) TaHsp90 docking to a predicted ligand (colored portion shows active site interaction with ligand). (C) Ramachandran plot showing the favourable region of TaHsp90 protein.

The cv. C306 *TaHsp90* was 99% similar to that of the *TaHsp90*.*2-B1* followed by *TaHsp90*.*2-A1* (98%) and *TaHsp90*.*2-D1* (98%) [31]. The predicted subcellular localization of TaHsp90 was in cytoplasm having the highest score of 3.767, followed by ER (Endoplasmic reticulum) and nucleus. The other predictable locations based on the score have been shown in Table 3. No transmembrane helix was observed in the TaHsp90 sequence. No signal peptide was detected in the TaHsp90 sequence from wheat cv. C306 in our study and it was found to have 59 predicted phosphorylating sites as shown in Fig 4. Protein functioning related to switch on and switch off depends on the posttranslational modification and the most important is phoshorylation of serine, threonine and tyrosine amino acid residues. Our TaHsp90 showed extensive phosphorylation on threonine and serine residues while tyrosine residues had less capability of phosphorylation (Fig 4A). We have found 14 different kinase specific prediction which are as follows: CKII (Casein kinase II), PKC (Protein kinase C), PKA (Protein kinase A), RSK (Receptor Tyr kinase), PKC (Protein kinase C), CKI (Casein kinase II), DNAPK (DNA dependent protein kinase), ATM (Ataxia-telangiectasia mutated), PKG (Protein kinase G), SRC (Sarcoma family kinases), INSR (Insulin receptor tyrosine kinase), EGFR (Epidermal growth factor receptor), cdc2 (Cell division cycle protein kinase) and GSK3 (Glycogen synthase kinase 3). The predicted secondary structure had 21 α-helices (39%), 19 β-strands (17%) and 40 coils (Fig 4B). The secondary structure was found to be 25% disordered. Percentage of amino acid composition having significant p value is shown in Fig 4c. N-linked glycosylation based on NetNGly server predicted 3 potent N-linked glycosylated sites, the results are shown in Table 4 (S6 Fig). Out of these N-glycosylated sites, position 370 having amino acid sequence NISR was most potent. For O-linked glycosylation, TaHsp90 was found to have only one potential site at position 562. TaHsp90 was 90% modelled, the best match of TaHsp90 was with the atomic cryoEM structure of *Homo sapiens* Hsp90-Cdc37-Cdk4 complex (PDB id: c5fwkA) (73% identical with 100% confidence, PDB info-signalling molecule, resolution-3.90 Å, Model dimensions (Å)- X:123.177, Y:82.328, Z:54.748) (Fig 5A). TaHsp90 was further docked to a molecule (predicted ligand) as shown in Fig 5B. Active site of TaHsp90 contains Asn39, Ala40, Ala43, Lys46, Asp81, Met86, Asn94, Arg100, Ser101, Gly102, Thr103, Gly120, Gln121, Phe122, Gly123, Val124, Gly125 and Phe126. These amino acids of TaHsp90 were involved in interacting with ligands [(AMP (Adenosine monophosphate), ADP (Adensine diphosphate), ATP (Adenosine triphosphate)]. The Ramachandran plot showed that 94.3% amino acid residues were in the favoured region, 4.2% amino acid residues were in the allowed region while 1.6% amino acid residues were in the outlier region (Fig 5C).

**Table 3:** Subcellular location of TaHsp90 as predicted by CELLO v.2.5 server.

Location	Score
Cytoplasmic	3.767
ER	0.794
Nuclear	0.223
Mitochondrial	0.046
Chloroplast	0.038
Cytoskeletal	0.036
Golgi	0.023
Extracellular	0.021
Vacuole	0.021
PlasmaMembrane	0.019
Peroxisomal	0.009
Lysosomal	0.003

**Table 4:** Potential N-linked glycosylated sites found in TaHsp90. The result was generated using NetNGlyc 1.2 server.

Position/Sequence	Potential	Jury agreement	N-Glyc result
39 NASD	0.6610	7/9	+
370 NISR	0.6839	9/9	++
428 NRTK	0.5500	7/9	+

**Fig 6:**
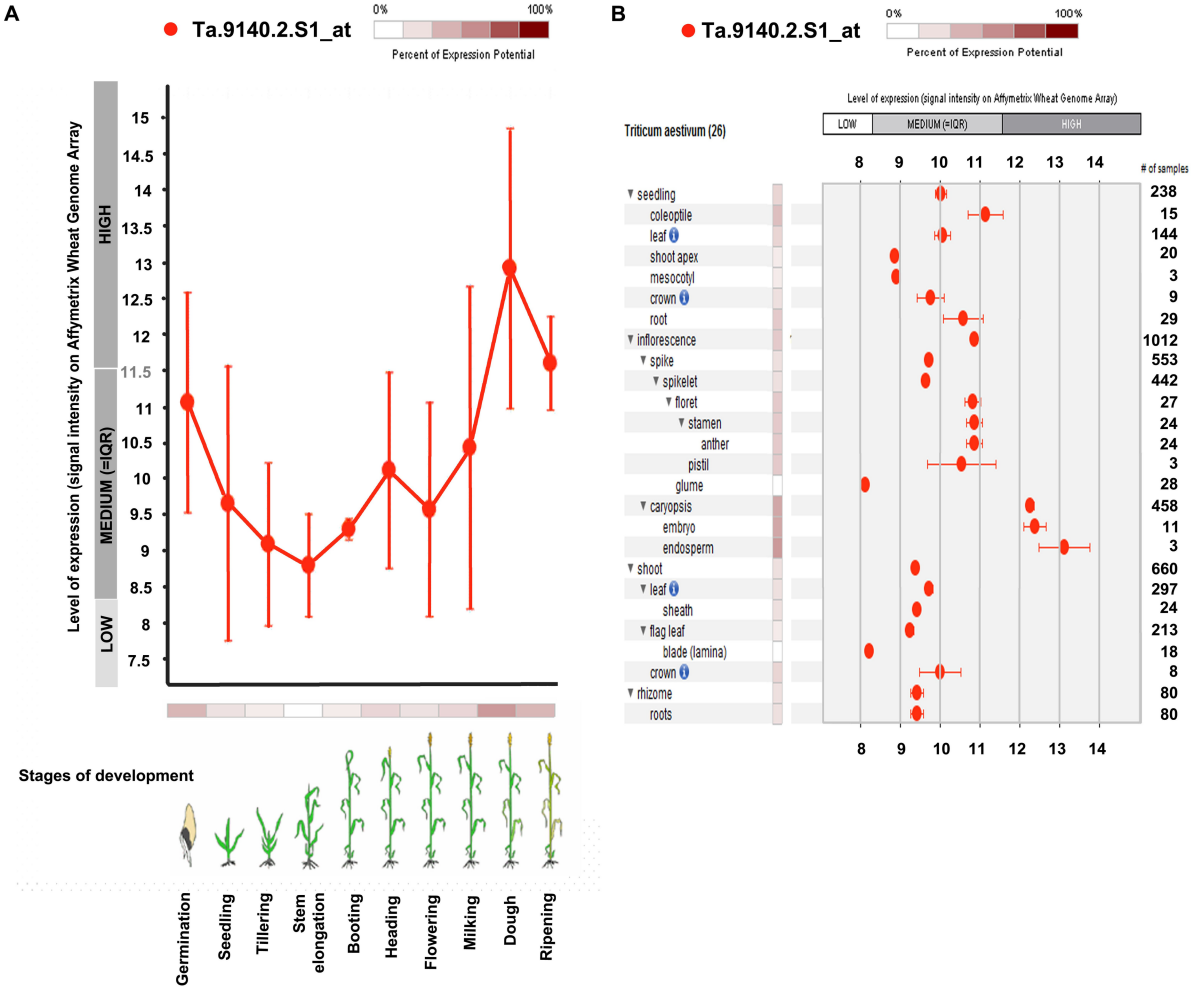
TaHsp90 digital expression in wheat. (A) TaHsp90 expression at 10 different stages of development (B) TaHsp90 expression at 26 anatomical parts of wheat tissue, key color code features given and # represents the number of samples in which the expression was observed. IQR stands for Interquartile range.

**Fig 7:**
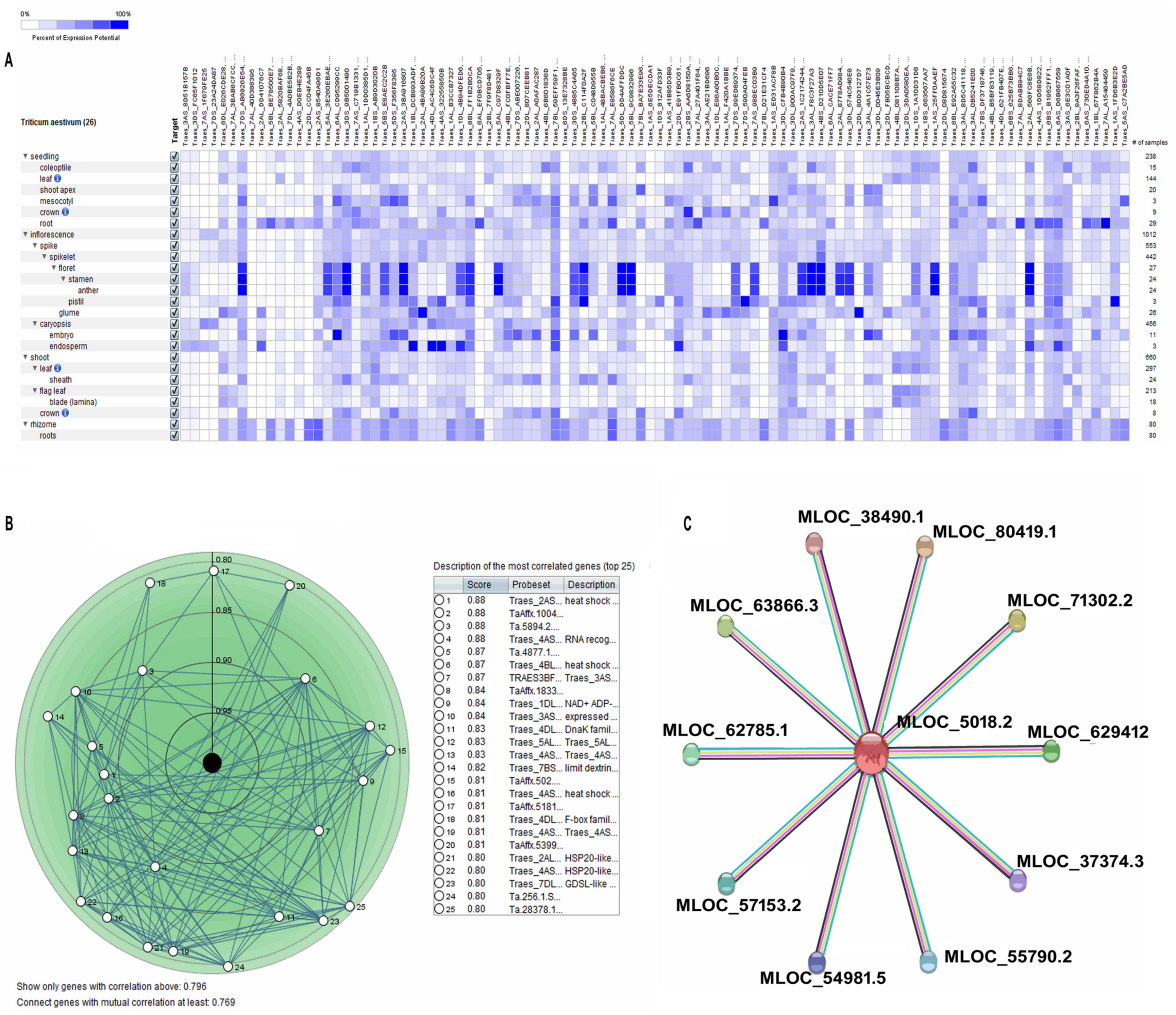
Tissue specific digital expression analysis of TaHsp90 with closely related genes. (A) Digital expression analysis of genes closely related to TaHsp90. (B) Pearson correlation coefficient analysis of TaHsp90 with closely related genes. (C) String based analysis of TaHsp90 for protein to protein interaction.

**Fig 8:**
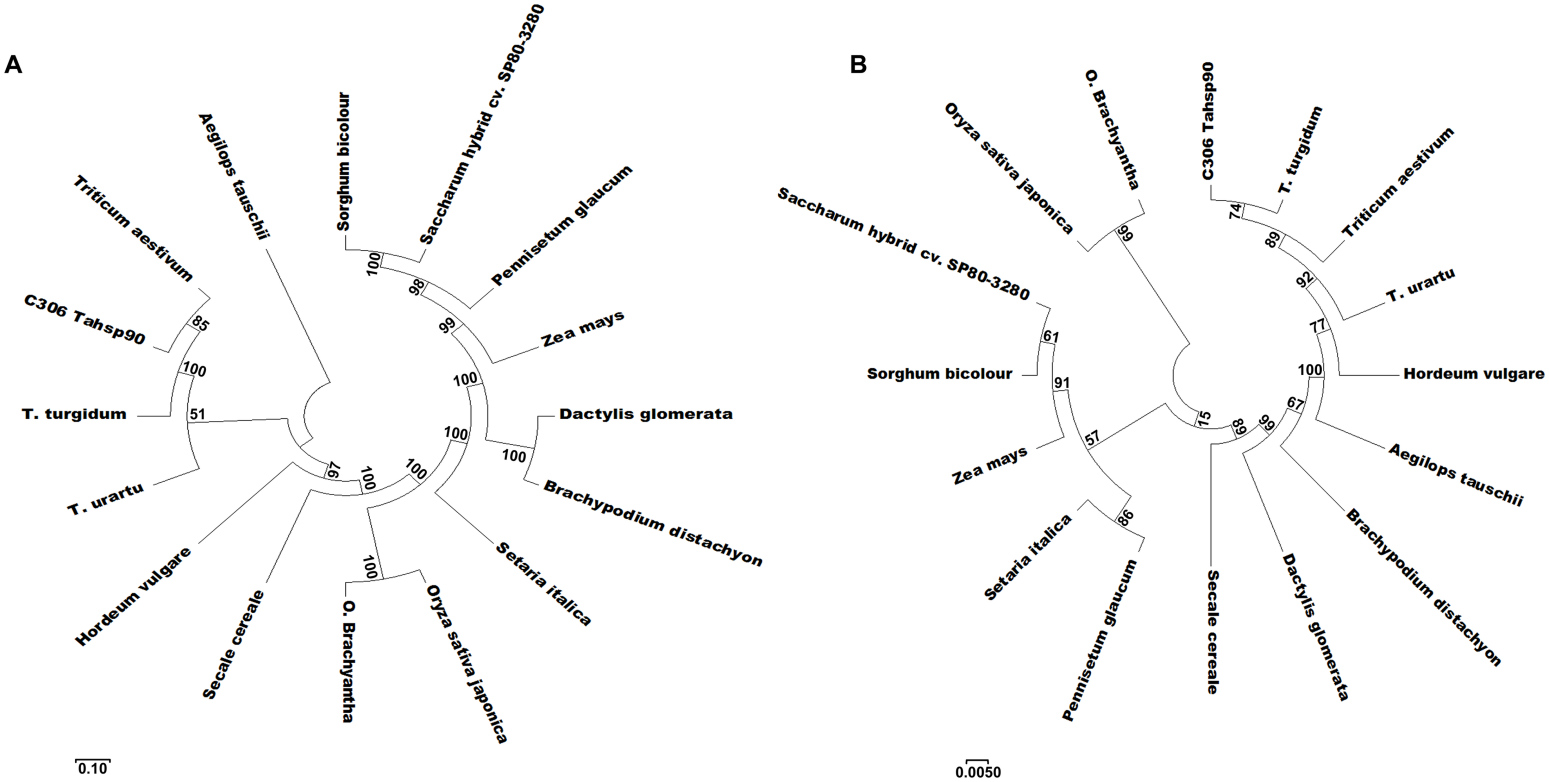
Evolutionay relationship of *T*. *aestivum* cv. C306 *TaHsp90* with other plant species. (A) Based on the nucleotide sequence. (B) Based on the amino acid sequence. Evolutionary analyses were conducted in MEGA7 using Neighbour-joining method with boot strap value of 1500.

The probe Ta.9140.2S1_at represents TaHsp90.1A1 in the Affymetrix wheat genome array. Its analysis under 10 developmental stages of wheat revealed that the highest expression was observed at the dough stage and lowest expression at stem elongation or jointing stage (Fig 6A). Level of expression was calculated based on Affymetrix wheat genome array data. It was observed that the expression started decreasing from germination to stem elongation stage and increased till dough stage with a slight fall at flowering stage. It was also observed that expression pattern was similar at seedling and flowering stage (between 9.5 to 10 folds in the medium range category). Similarly, at anatomical level, among the 26 tissues of wheat, highest expression was observed in the endosperm tissue (13 folds) while the lowest expression was observed in the glume (8 folds) (Fig 6B). It was observed that expression was similar in seedling, leaf and crown tissues (10 folds, in the medium range category). Similar expression pattern was also observed in the inflorescence, floret, stamen and anther tissues. Embryo and caryopsis showed almost similar level of expression in the high range category (12–12.5 folds). The digital tissue specific expression analysis showed marked differential expression among closely related genes (probesets). Most of the probesets were highly expressed in floret, anther, stamen and endosperm tissue (Fig 7A). The Pearson correlation coefficient is shown in Fig 7B. The table given along with figure describes different probesets most correlated with TaHsp90, having a top score of 0.88 and minimum score of 0.80. The digital expression pattern of gene *Hsp90* in *Oryza sativa* showed expression in the high range category (~ 17 folds) throughout all the stage of development except during panicle differentiation stage. Also, the gene *Hsp90* of *Zea mays* and *Hordeum vulgare* were in the high range category. The expression pattern was found to be most variable in the *Hordeum vulgare* as compared to *Oryza sativa* and *Zea mays* (S7 Fig). String based protein to protein interaction has been shown in the Fig 7C, which was based on *Hordeum vulagare*, due to its close relatedness with *Triticum aestivum* (based on phylogenetic analysis). An unrooted phylogenetic tree was constructed, revealed the evolutionary distance of *TaHsp90* from wheat cv. C306 of the present study with that of other plant species (Fig 8A and 8B). It was found that, it was closely related to Hsp90 of *Tritcum turgidum* followed by *Triticum urartu and Hordeum vulgare*.

### *TaHsp90* overexpression in *E*. *coli*, protein gel blot, His-tag purification, MALDI-TOF analysis and heat stress tolerance assay

**Fig 9:**
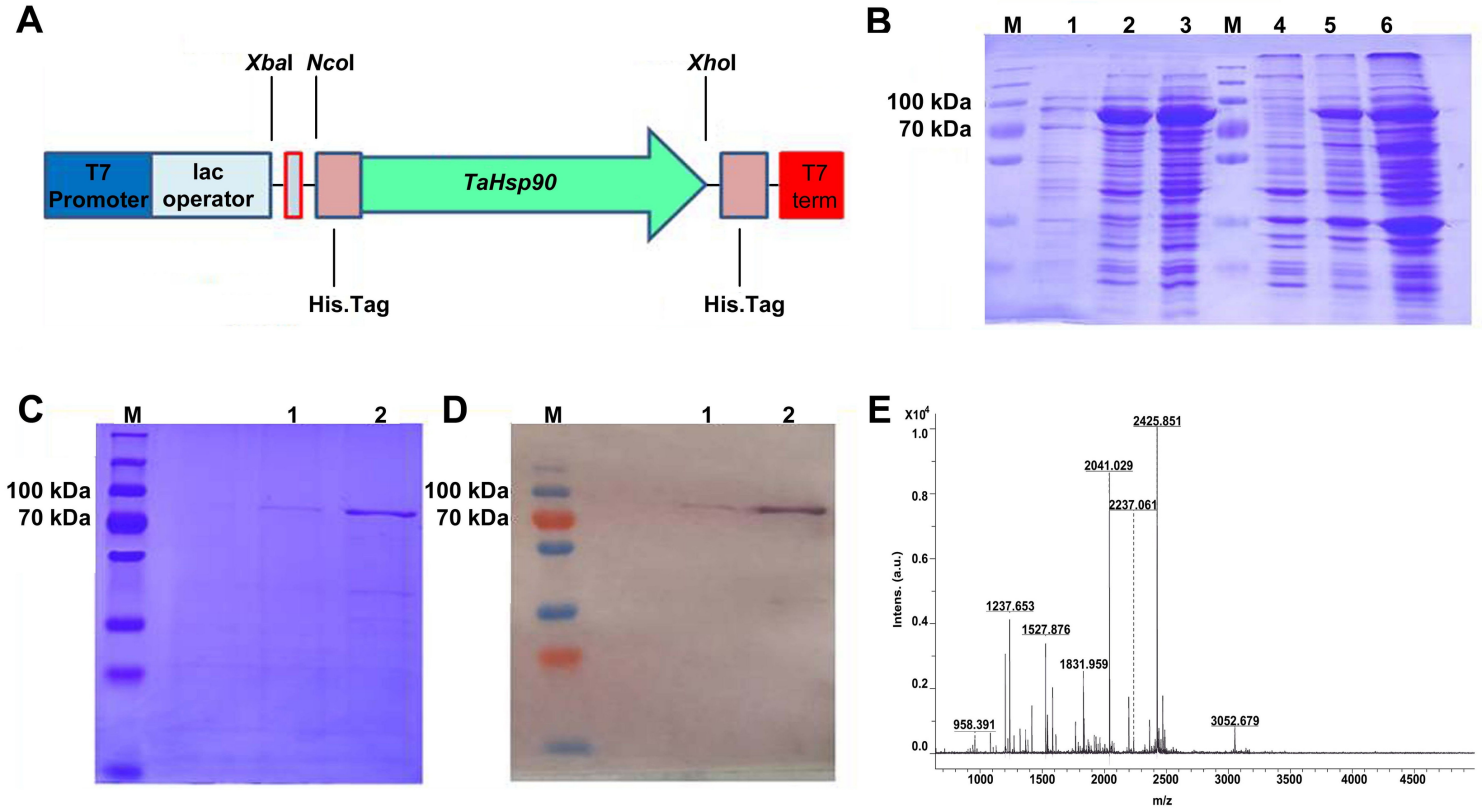
Overexpression of *TaHsp90* in prokaryotic model system. (A) pET28-*TaHsp90* construct shows main features of the construct. (B) Overexpression of TaHsp90 protein on SDS-PAGE. (C) Purification by protein by His-pull down assay using Ni-NTA columns. (D) Protein gel blot analysis using anti his-tag antibody. (E) Mass spectra obtained after MALDI-TOF analysis of single purified band, X-axis represents mass/charge ratio, Y-axis represents Intensity.

The pET28-*TaHsp90* construct in BL21 cells showed a prominent overexpressed band between 70 kDa and 100 kDa marker (Fig 9A and 9B). The His-tag affinity purified protein sample was also observed at ~80 kDa as in the expected manner (Fig 9C). The protein gel blot carried out with Anti-His-tag antibody also revealed the developed band at the same location (Fig 9D). The MALDI-TOF analysis of the purified band further confirmed the TaHsp90 protein with a top score of 165, protein score greater than 90 were considered significant (*p*<0.05) (Fig 9E) (S1 File). The best match was with accession no. 294717812 having molecular mass of 80.69 kDa which was in conformity with the theoretical molecular mass of our TaHsp90 protein (80.425 kDa). The CFU/mL under controlled and heat stress conditions for *E*. *coli* BL21 cells carrying construct pET28- *TaHsp90* and pET28 vector alone has been shown in (S8 Fig). It was observed that *E*. *coli* BL21 cells carrying construct pET28-*TaHsp90* were found to tolerate high temperature (45°C) stress as compared to cells having pET28 vector alone.

## Discussion

Wheat is one of the most important cereal crops of the world, which is being adversely affected due to increasing global warming. Considering the importance of wheat-heat interaction, insights are needed into genetic and molecular mechanisms that control stress responsive metabolic pathways in heat tolerant cv. C306.

Here, we combined SSH, qRT-PCR and reverse northern dot blot analysis to identify the potential genes that are differentially expressed in response to heat stress (HS) with the aim to investigate molecular mechanism underlying for HS tolerance in wheat cv. C306. Secondary amplification of cDNA synthesis was visualized as a smear in the range of 100 bp to 1.5 kb on 1.2% agarose gel which indicated successful library preparation, and this result was in uniformity with Gurjar et al. [11]. For identifying and enriching differentially expressed genes under HS in wheat cv. C306, SSH has been an efficient approach in various crops [11–13]. We constructed two HS responsive forward SSH libraries (37°C HS and 42°C HS) at post anthesis stage in heat tolerant bread wheat cv. C306. 1728 EST were assembled to 403 singlets and 175 contigs. This information enriches wheat genome databases and provides basic molecular biology information for gene cloning and analysis of HS responsive metabolic pathways. ESTs obtained in this study showed a clearly defined coupling of plant stress tolerance under HS, which can be further used as HS tolerance linked markers. Under molecular function category the sequences belong to “protein binding and catalytic activity” were found to be most abundant (43 sequences each) under 37°C of HS. These results were in uniformity with that reported by Padaria et al. [32]. 15.2% contigs and 26.87% singlets did not show any homology, these sequences will add further genomic resource to wheat data base and can be utilized for finding uncharacterized genes/transcription factors etc. These unknown/novel genes or functionally uncharacterized genes would provide a candidate genetic resource for abiotic stress tolerant genes. A chromosome based draft sequence of wheat genome (*T*. *aestivum* cv. Chinese spring) is available, in which 1,24,201 annotated gene loci showed uniform distribution across the subgenomes they have also shown the high sequence similarity of hexaploid wheat to that of diploid and tetraploid ancestors [1]. This is the first study related to HS responsive SSH library preparation of Indian wheat cv. C306 at post anthesis stage and the data has been submitted to EMBL-EBI (Accession no. LT965080 to LT965927: 37°C forward HS library; LT963784 to LT964663: 42°C forward HS library). All the analyzed transcripts have a distinct and important role in activating the tolerance mechanisms under heat and other abiotic stresses. 1728 ESTs were mapped to a total of 55 metabolic pathways (KEGG pathways) which were affected by HS treatment. In this way, an alternative functional annotation to ESTs was provided associated to their biochemical pathway. 11.3% and 30.77% ESTs from 37°C and 42°C HS SSH libraries, respectively, did not show any match to the existing database of NCBI.

**Fig 10:**
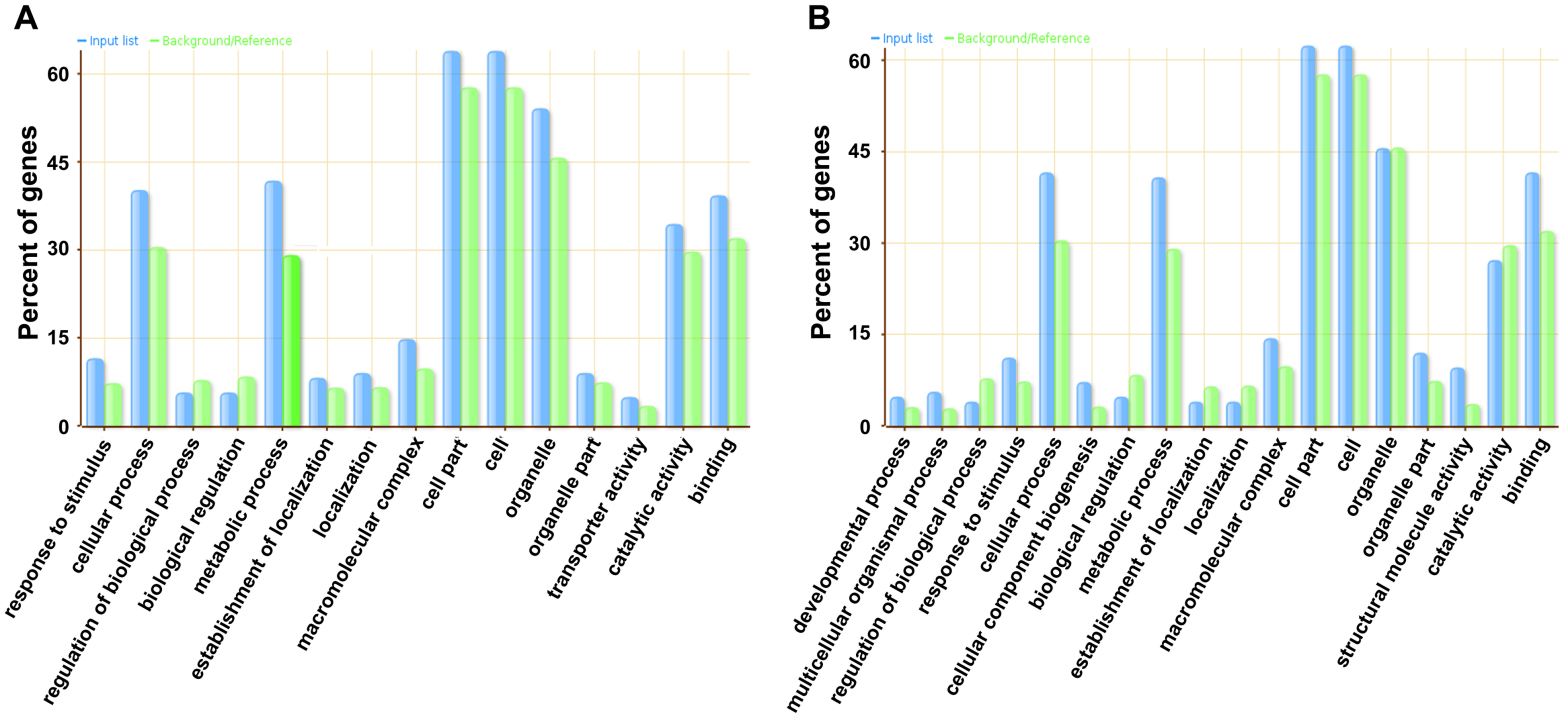
Heat inducible changes in Indian wheat cv. C306 under heat stress as detected by SSH approach.

Validation of differential expression of ten selected genes under HS was carried out in heat tolerant (cv. C306) and heat susceptible (cv. HD2967) wheat cultivars. The specificity of qRT-PCR amplification was confirmed by a single peak during melting temperature curve analysis of qRT-PCR amplified products. *β-actin* was used as internal control [24, 29]. Further, three candidate genes code for *Hsp-Sti*, *Hsp90*, hypothetical *Dnaj* were also studied at different time intervals (30min, 1h, 2h, 4h, 6h) of HS in tolerant wheat cv. C306, confirming their role in early HS response. Higher level of GPX gene expression in wheat cv. C306 suggests that the potential role of antioxidant machinery for combating HS response. Antioxidant enzymes such as SOD, APX, CAT, GPX works in combination at the time of HS to decrease the level of antioxidants/ROS (Reactive Oxygen Species) such as H_2_O_2_, ^•^O_2_^-^. Upregulation of ESTs showing homology to Metallothionenins or MTs was observed to be high confirming their role in HS tolerance. Studies by previous workers have also reported the role of metallothioneins in scavenging ROS [33–35]. PPIase expression was observed to be significantly higher (3.03 folds at 42°C) in wheat cv. C306 as compared to its expression in cv. HD2967 (0.3 folds at 42°C). Enzyme PPIase contains amino acid proline as its main component and it basically functions as a chaperone for proper protein folding under HS, which was in conformity with the findings of Goswami et al. (2016) [21]. Kurek et al., 1999 have reported that the expression of FKBP77 (encoding PPIase) in wheat cv. Atir was 14 folds higher under HS at 37°C, which defined it as heat stress dependent isoform [36]. *Hsp-Sti* observed in this study showed the highest fold change (76.62 folds) expression among observed ESTs, however, exact biological role of these stress-inducible (Sti)-Hsps has not yet been elucidated [37]. It is also reported that heat stress inducible proteins accumulated more in thermotolerant perennial creeping bentgrass (*Agrostis scabra*) as compared to heat susceptible species of grass, *Agrostis stolonifera* [38]. These stress inducible-Hsps can be further utilized for their potential role in HS response. Heat inducible genes were upregulated in transgenic plants overexpressing heat shock related transcription factor A (HsfA), transgenic *Arabidopsis* overexpressing HSFA2 could rescue defects of mutant (HSFA1 QK mutant) under heat stress conditions [39]. CCDs (Carotenoid cleavage dioxygeneases) are non-heme important signalling molecules such as abscisic acid (ABA) and strigolactone, further ABA plays important role in drought and HS tolerance, seed development and sugar signalling pathway. Very little information is available regarding molecular mechanism of CCDs, however CCD4 isoform from *Crocus sativa* was significantly induced by heat and dehydration stress treatment [40]. Further, an important co-chaperone of Hsp70, ss*Dnaj* plays an important role in assembly, protein folding and unfolding under stress conditions. Here putative *Dnaj* from wheat cv. C306 was upregulated (2.8 folds) under HS whereas susceptible cv. HD2967 showed fold change of only 0.8 fold. However, in this study several stress marker genes such as *dhn*, *dun*, *wcor* were not isolated from any of the two HS subtracted library. The possible reason could be that only limited number of clones were picked up, time duration of HS, presence/absence of these genes in two forward SSH libraries. Further, heat stress inducible changes in Indian wheat transcriptome under high temperature as detected by SSH approach has been shown in Fig 10.

Margaritopoulou et al. reported that moderate HS treatment increased Hsp90 mRNA expression levels in *Arabidopsis* and helped in flowering [41]. In plants, RAR1-SGT1-Hsp90 [(RAR1-Required for Mla12 (mildew resistance locus A) Resistance 1 and SGT1 is Suppressor of G2 allele of *skp*1 (suppressor of kinetochore protein)] complex binds in 1:1:2 ratio and confers tolerance to stress. The same complex is formed in animal cells at the time of immune response and is known to confer immunity against pathogens [42]. In this work, *TaHsp90* was selected as a candidate gene for genomics, proteomics and *in-silico* based studies. Our results predicted that TaHsp90 was mostly found in cytoplasm with a CELLO prediction score of 3.767 which suggests its highly soluble and hydrophilic nature. Members of Hsp90 family possess penta-peptide MEEVD C-terminus which is a characteristic of cytosolic Hsp90 isoforms [43]. No transmembrane helix nor signal peptide was found in the TaHsp90 sequence. This could be one of the reasons for TaHsp90 not being targeted to any particular organelle. Wang et al. also reported nine cytosolic *TaHsp90* genes which were shown to be located on three different chromosomes i.e., 2^nd^, 5^th^ and 7^th^ chromosome. They also reported that the presence of two introns in the genomic sequence of *TaHsp90* were responsible for variation in the length [31]. Our TaHsp90 had 59 phosphorylating sites, this result was in contrast to that found by Kumar et al. which had shown 39 phosphorylating sites [44]. Phosphorylation capabilities of serine and threonine residues were more as compared to tyrosine and this finding was in uniformity with Basharat Z., who had predicted phosphorylation in Hsp70 of *Triticum aestivum* [45]. These kind of phosphorylated proteins are usually targeted to chloroplasts [46]. Site directed mutagenesis of important phosphorylated sites would elucidate the role of TaHsp90 under abiotic stress [47]. Many cell metabolic activities like RNA binding, splicing activity, protein-protein interaction, subcellular localization are influenced by phosphorylation. Zhang and Mount showed that two alternatively spliced isoforms of *Arabidopsis* SR45 protein (Ser-Arg rich) have different biological functions due to mutation caused at predicted phosphorylation site [48]. A refined 3D structure of TaHsp90 was generated successfully which can be used for understanding function at molecular level and active sites identification. However, structural significance of full length Hsp90 have been shown in bacteria, yeast and mammals [49–51]. We report here the secondary and 3D structure prediction of TaHsp90 which provides information related to active sites prediction and basic molecular functioning of the protein. Using PDB-PSI-BLAST (https://www.ebi.ac.uk/Tools/sss/psiblast/), TaHsp90 maximum identity deciphering was at 73% which is considered to be good score to start modelling. 3D structure was further verified by Ramachandran plot and it showed that 93.4% residues were in favoured region, validating the 3D protein conformation of TaHsp90. Further, digital expression of TaHsp90, and its close association with other genes was carried out. TaHsp90 digital expression at different developmental stages of wheat was mostly in the interquartile range (medium range) and similar pattern was observed for tissue specific expression pattern in 26 different anatomical parts. Developmental stage specific and tissue specific based expression pattern of TaHsp90 would be useful for selecting the wheat stage and tissue for qRT-PCR expression, transcriptome and molecular marker based studies. It was also found that the digital gene expression analysis revealed dynamic temporal expression of Hsp90 during different developmental stages of wheat, rice, maize and barley. The expression of Hsp90 remained relatively constant throughout the life cycle of rice and maize. The gene expression was found to be highest during flowering stage in barley while it showed attenuation in expression at the time of flowering in wheat. We speculate that this gene might be involved in multiple genetic pathways in involved in different developmental stages of crops especially flowering. Gene-specific expression provide evidence of when and where gene products may function and provide insights to the involved metabolic pathways which supports approaches for functional investigation through reverse genetics. Computational based studies of TaHsp90 would have greater practical significance not only related to its structure but also related to post translational modifications. Goswami et al. [21] used different software tools available on server of GPS-polo 1.0 (http://polo.biocuckoo.org/) for predicting the post translation modification sites (PTMs) and concluded that these PTMs may be used in manipulating the expression of genes/proteins associated with thermotolerance. Until now there is no report of cryo-atomic electron microscopic structure of Hsp90 from wheat, studies related to these would further help in understanding structural functioning and interaction with other molecules. Here, *TaHsp90* was validated for overexpression in a prokaryotic system, *E*. *coli* BL21 (DE3). The overepxressed TaHsp90 protein imparted tolerance to *E*. *coli cells* grown under HS conditions, confirming its role in HS response. There are reports in which genes from different plant species e.g. *Dreb* from *Salicornia brachiata*, *Phytochelatin synthase* from *Anabaena* sp., *LEA* gene from soybean, *NAC4* transcription factor from horsegram, *Asr1* from *Ziziphus nummularia* have been validated for overexpression in *E*. *coli*. [52–56]. However, we reported *TaHsp90* from Indian wheat cv. C306, overexpression in *E*. *coli* BL21 (DE3) cells. Functional validation of gene from plant systems in prokaryotic system are reported. *TapAPX* gene from heat tolerant cv. Raj3765 when cloned in an expression vector and transformed to *E*. *coli*., was able to provide HS tolerance to *E*. *coli* cells at elevated temperature [29]. Similarly, overexpression of *Hsp90* is known to provide abiotic stress tolerance in transgenic plants. Overexpression of *Glycine max Hsp90* was able to confer abiotic stress tolerance in transgenic *Arabidopsis* [17]. *Arabidopsis thaliana Hsp90* is coded by seven genes, of which *AtHsp90*.*1* is highly stress inducible and *AtHsp90* can be specifically inhibited by treating with geldanamycin (GDA) to study morphological abnormalities [57]. Similarly, Samakovll et al (2007), shown the importance of *Hsp90* in knock out lines lacking *AtHsp90* gene, these lines were lacking proper growth and were observed with other abnormalities like uneven growth of cotyledons, long hypocotyls, rootless seedling and triplet cotyledons [19]. The study of *TaHsp90* in prokaryotic system as well as model plant systems will provide insights to its role under HS conditions and HS tolerance. *TaHsp90* can be taken up as a candidate gene for developing heat tolerant crop plants under the control of constitutive or stress inducible promoter either through marker assisted breeding or transgenic approach for a climate resilient agriculture.

## Supporting information

S1 Fig Average length distribution of heat stressed ESTs. (TIF)

S2 Fig Distribution of enzyme classes of HS libraries. (TIF)

S3 Fig GO analysis of heat stressed ESTs in three categories. (TIF)

S4 Fig Interactive map plot of SSH libraries using REVIGO. (TIF)

S5 Fig ClustalW alignment of TaHsp90 with Hsp90 of other plant species. (TIF)

S6 Fig N-linked and O-linked glycosylation potential of TaHsp90. (TIF)

S7 Fig Comparative digital expression analysis of *TaHsp90* to closely related species. A-*Triticum aestivum*. B-*Oryza sativa*. C-*Zea mays*. D-*Hordeum vulgare*.
(TIF)

S8 Fig Heat stress tolerance assay in *E*. *coli* BL21 cells. Statistical significance has been shown by asterisk (*) at p≤0.05 (n = 3).
(TIF)

S1 File Mascot search analysis of TaHsp90 purified protein expressed in *E*. *coli*. (PDF)

S1 Table Enriched biological processes related GO terms under HS at 37°C and 42°C. (DOCX)
