# Supplementary figures and images for: Heat stress transcripts, differential expression, and profiling of heat stress tolerant gene TaHsp90 in Indian wheat (Triticum aestivum L.) cv C306
Source: PLoS One. 2018 Jun 25;13(6):e0198293. doi: 10.1371/journal.pone.0198293 (PMC6016904; doi:10.1371/journal.pone.0198293)

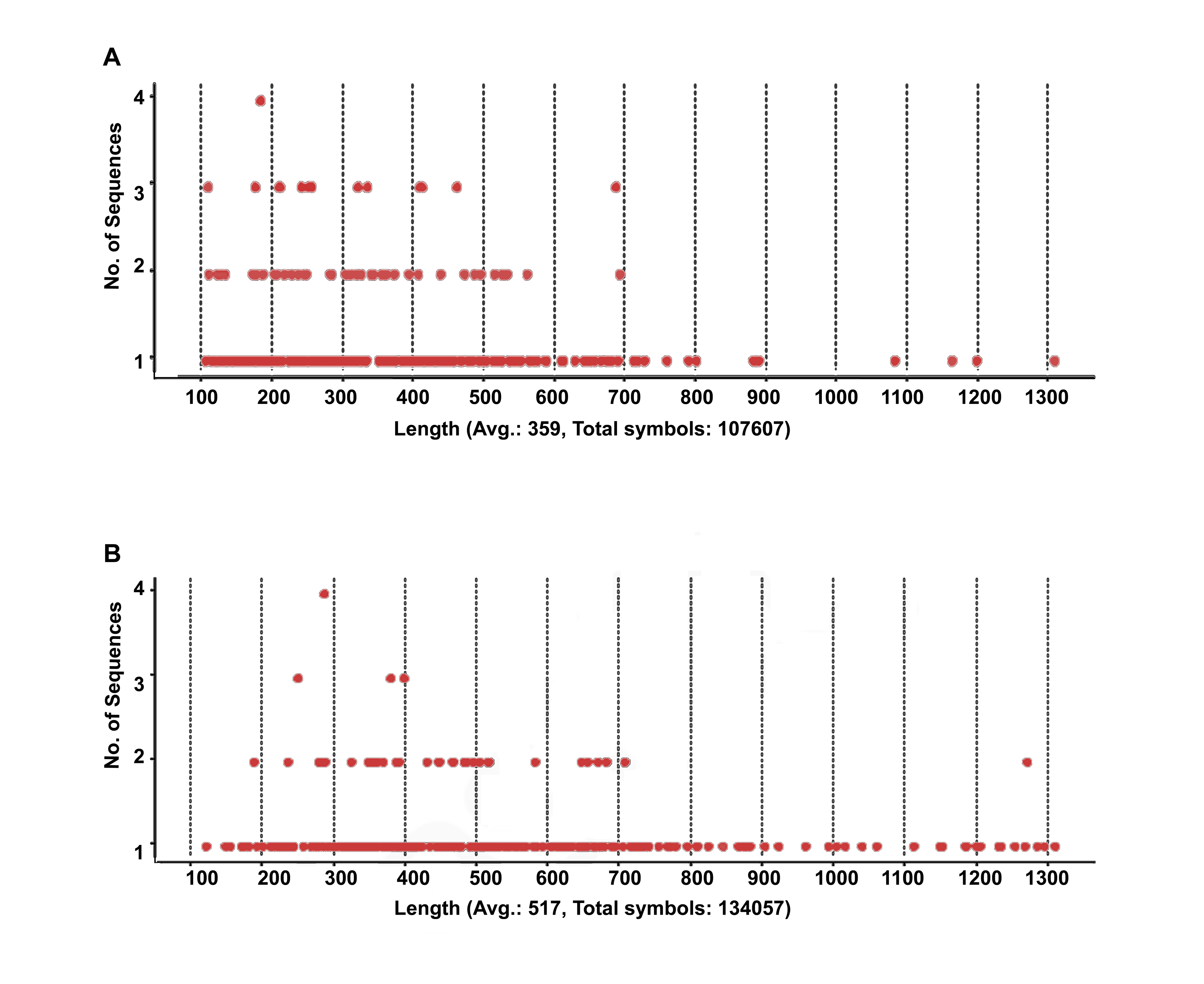

Supplement: S1 Fig — (TIF) [file pone.0198293.s001.tif]

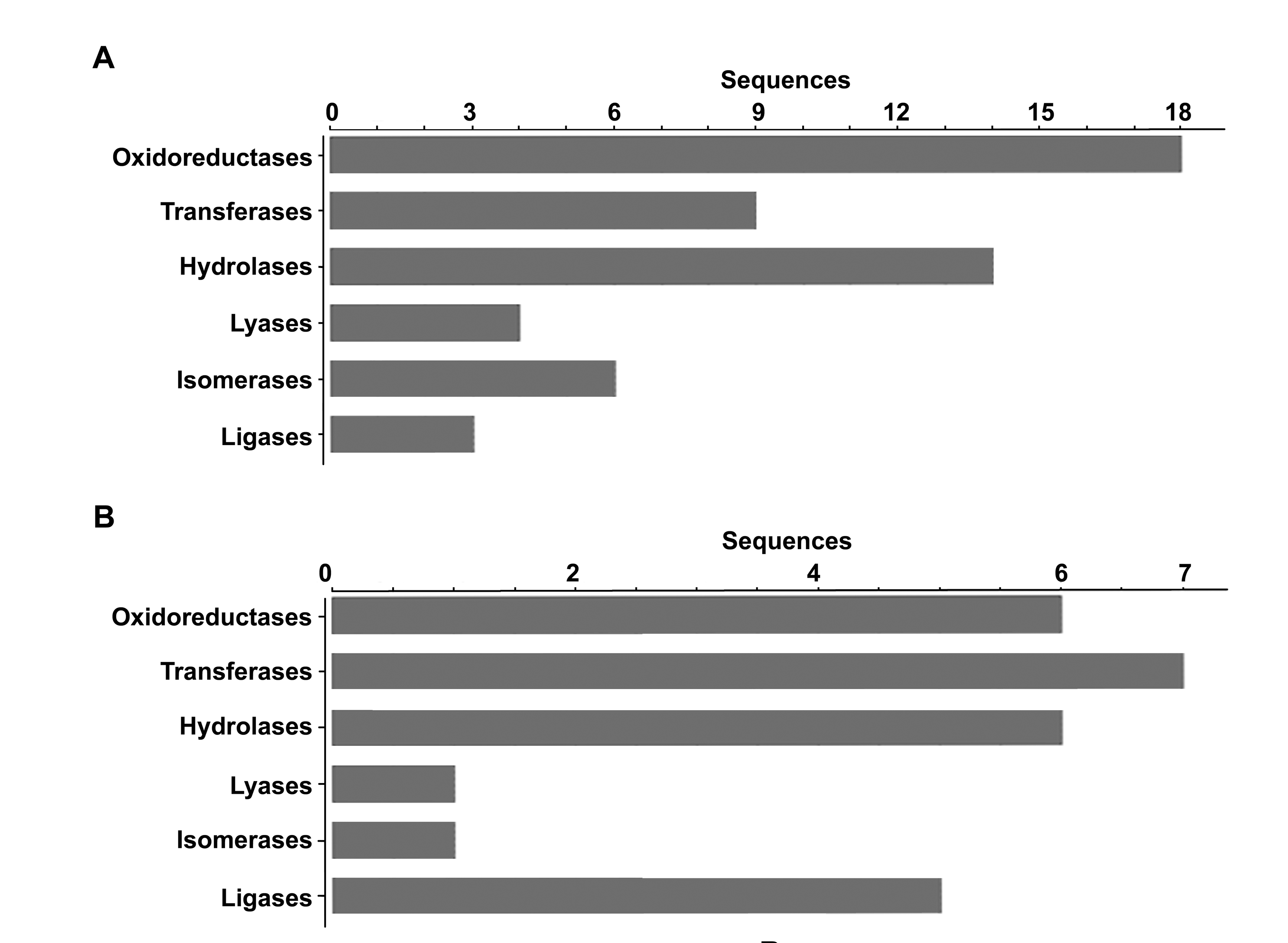

Supplement: S2 Fig — (TIF) [file pone.0198293.s002.tif]

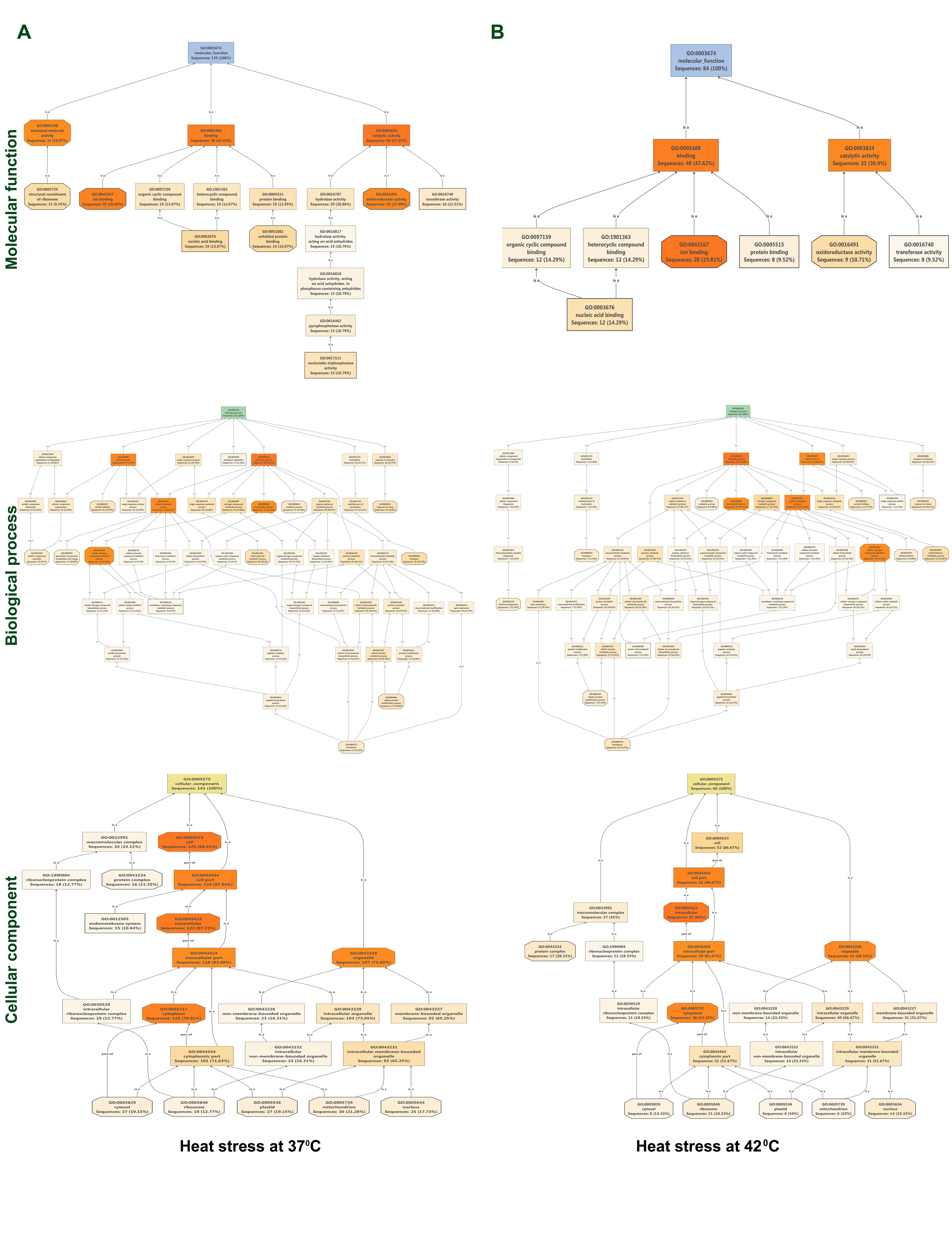

Supplement: S3 Fig — (TIF) [file pone.0198293.s003.tif]

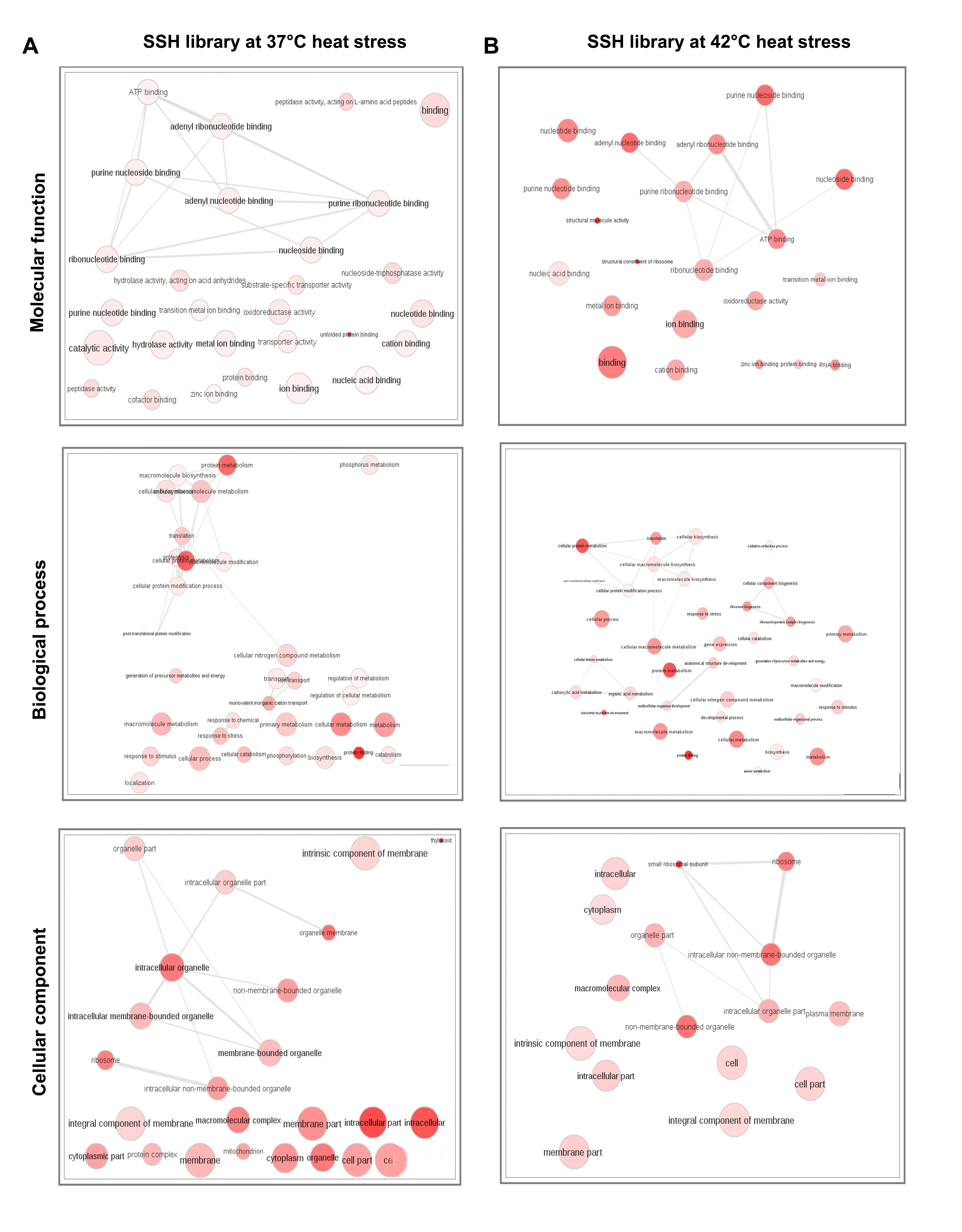

Supplement: S4 Fig — (TIF) [file pone.0198293.s004.tif]

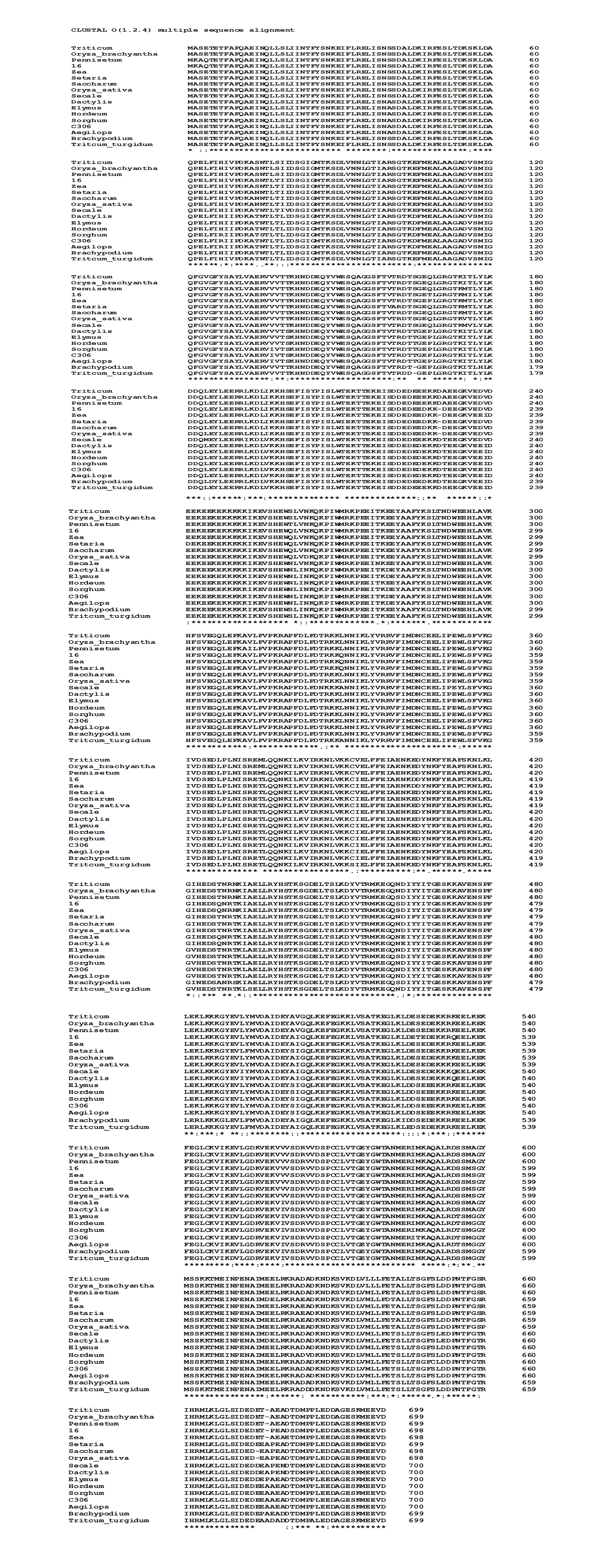

Supplement: S5 Fig — (TIF) [file pone.0198293.s005.tif]

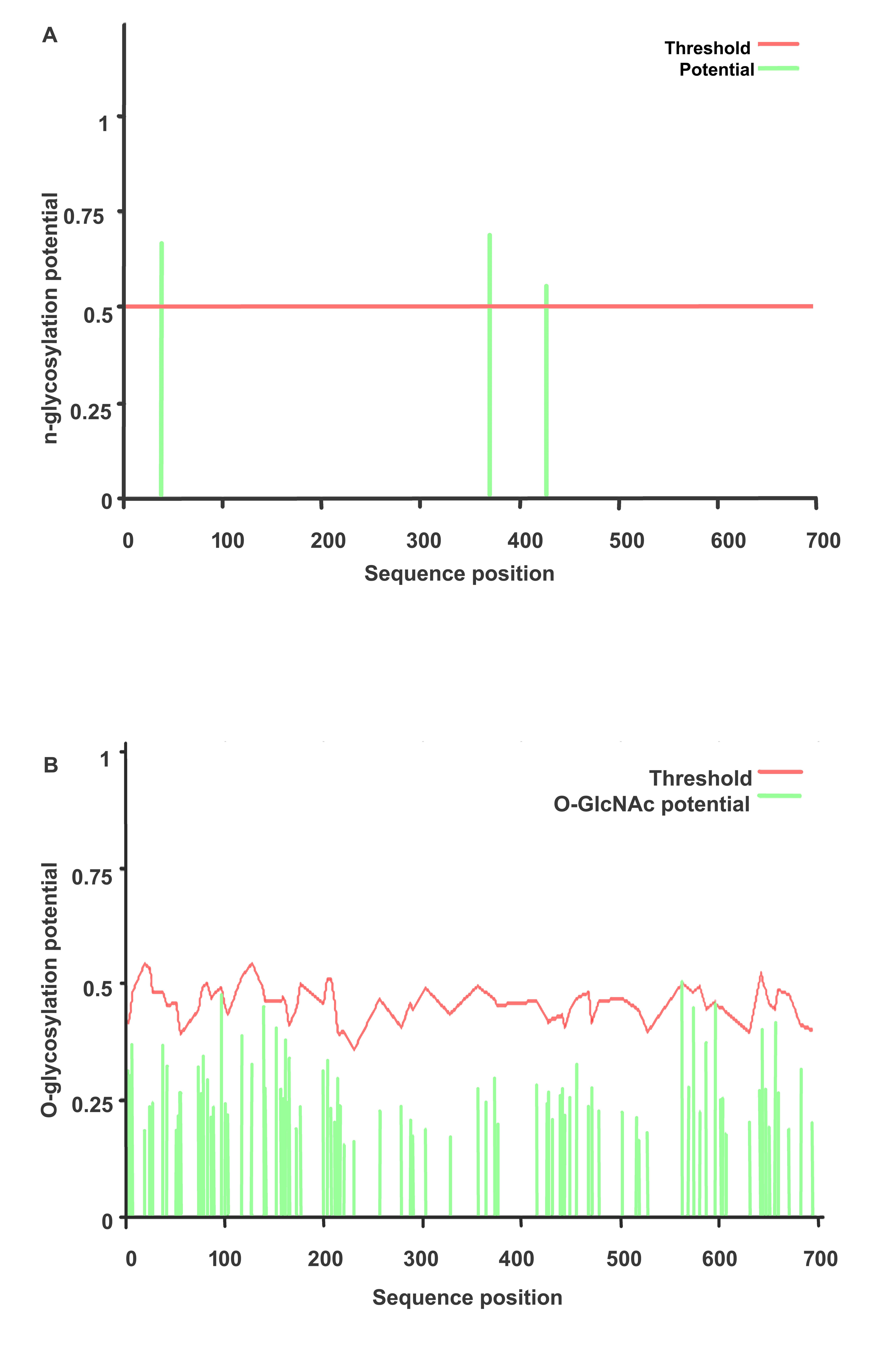

Supplement: S6 Fig — (TIF) [file pone.0198293.s006.tif]

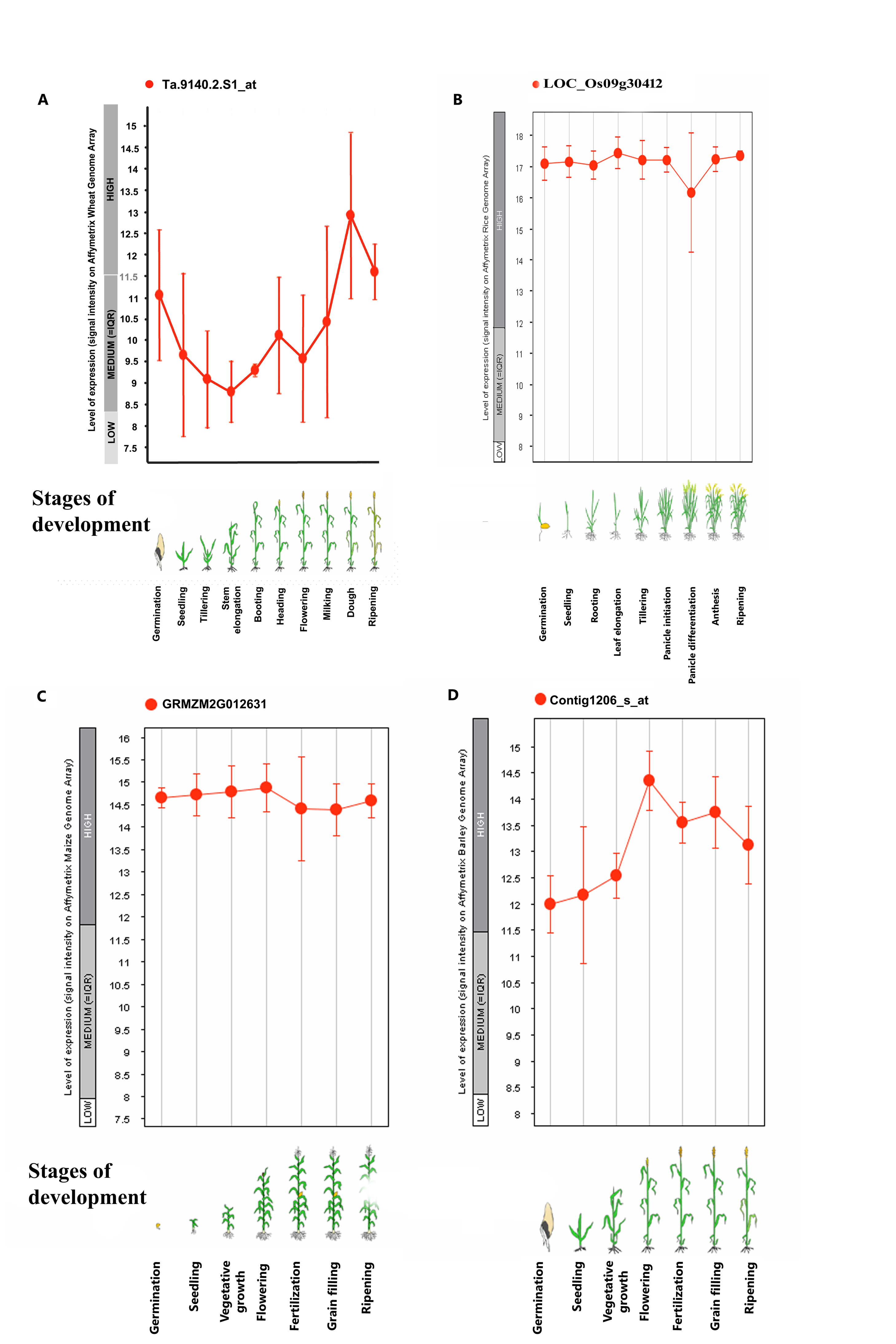

Supplement: S7 Fig — A-Triticum aestivum. B-Oryza sativa. C-Zea mays. D-Hordeum vulgare. (TIF) [file pone.0198293.s007.tif]

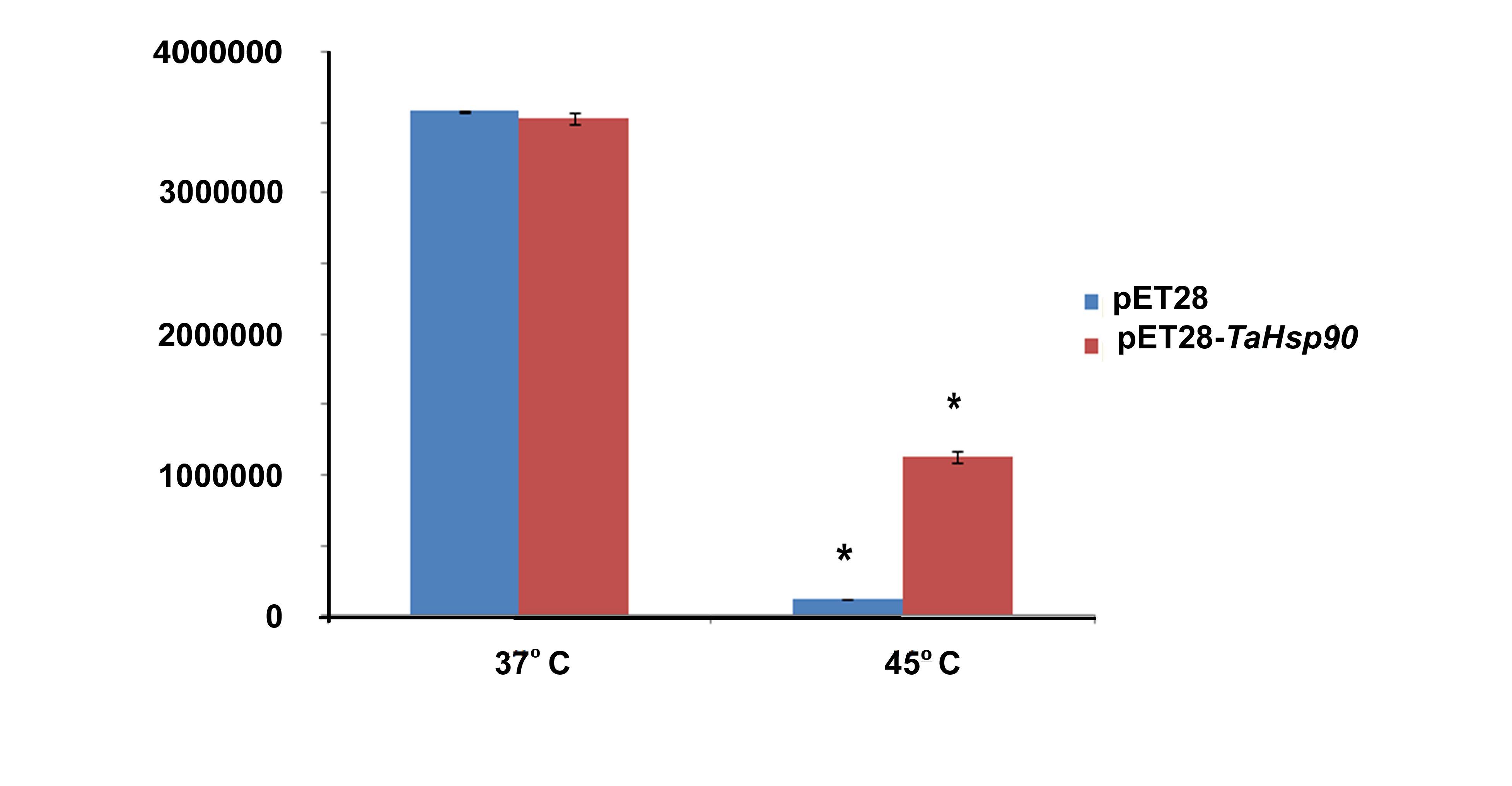

Supplement: S8 Fig — Statistical significance has been shown by asterisk (*) at p≤0.05 (n = 3). (TIF) [file pone.0198293.s008.tif]
