# Supplementary material for: Heat stress transcripts, differential expression, and profiling of heat stress tolerant gene TaHsp90 in Indian wheat (Triticum aestivum L.) cv C306
Source: PLoS One. 2018 Jun 25;13(6):e0198293. doi: 10.1371/journal.pone.0198293 (PMC6016904; doi:10.1371/journal.pone.0198293)
Supplement: S1 File — (PDF) [file pone.0198293.s009.pdf]

Mascot Search Results

```
User               : saroja
Email              : roja.295@gmail.com
Search title       :
Database           : NCBInr 20150509 (66387522 sequences; 23805201081 residues)
Timestamp          : 14 May 2015 at 05:39:23 GMT
Top Score          : 165 for gi|294717812, heat shock protein 90 [Triticum aestivum]
```

Mascot Score Histogram

Protein score is -10*Log(P), where P is the probability that the observed match is a random event.
Protein scores greater than 91 are significant (p<0.05).

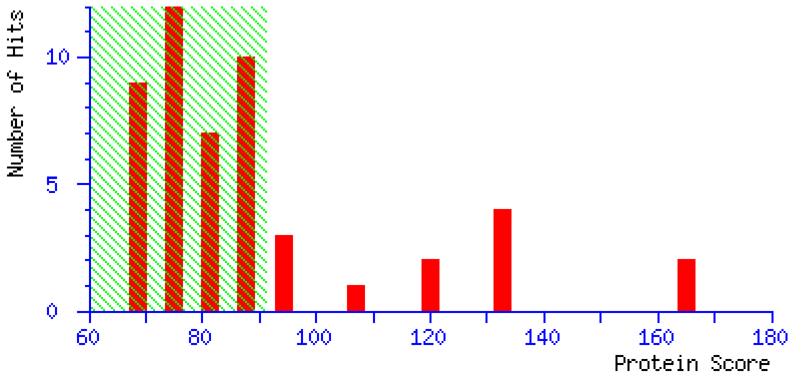

Protein Summary Report

Format As | Protein Summary | Help

Significance threshold p< 0.05 | Max. number of hits 20

Preferred taxonomy All entries

Overview Table

Click on column header to jump to entry in results list.
Move mouse over any indicator to highlight identical peptides.
Click on an indicator to see details of individual match.
Use check boxes to select sub-set of queries for new search.

Mouse over: -Query-

-Accession- -Sequence-

Hit:	1	2	3	4	5	6	7	8	9	10	11	12	13	14	15	16	17	18	19	20
☑ 941.3557 (1+)																				
☑ 958.3910 (1+)																				
☑ 1081.4788 (1+)	●	●	●	●	●	●	●	●	●	●	●	●	●	●	●	●	●	●	●	●
☑ 1127.5139 (1+)	●	●	●	●	●	●	●	●		●										
☑ 1201.6596 (1+)	●	●						●										●		
☑ 1222.7685 (1+)																				
☑ 1237.6534 (1+)	●	●	●	●	●	●	●	●	●	●	●	●	●	●	●	●	●	●	●	●
☑ 1272.7172 (1+)	●	●	●	●	●	●	●	●	●	●	●	●	●	●	●	●	●		●	●
☑ 1320.7004 (1+)	●	●	●	●	●	●	●	●	●	●	●	●	●	●	●	●	●	●	●	●
☑ 1365.7740 (1+)																				
☑ 1384.8601 (1+)																				
☑ 1416.8454 (1+)	●	●						●										●		
☑ 1527.8755 (1+)	●	●	●	●	●	●	●	●	●	●	●	●	●	●	●	●	●	●	●	●
☑ 1541.8429 (1+)	●	●	●	●	●	●	●		●	●	●	●	●	●	●	●	●		●	●
☑ 1544.9082 (1+)	●	●	●	●	●	●	●	●	●		●	●		●				●		
☑ 1583.8678 (1+)	●	●	●	●	●	●	●	●	●	●	●	●	●	●	●	●	●	●	●	●
☑ 1609.9427 (1+)	●	●	●	●	●	●	●	●	●			●	●					●		●
☑ 1768.9015 (1+)																		●		
☑ 1789.9088 (1+)	●	●	●	●	●	●	●	●		●					●	●	●		●	●
☑ 1831.9592 (1+)	●	●	●	●	●	●			●			●		●						
☑ 1836.9618 (1+)	●	●	●	●	●	●	●	●	●	●	●	●	●	●	●	●	●		●	●
☑ 1869.9439 (1+)																				
☑ 1917.9641 (1+)	●	●	●	●	●	●	●	●		●					●	●	●		●	●
☑ 1933.9549 (1+)	●	●	●	●	●	●	●	●	●	●	●	●		●	●	●	●	●	●	●
☑ 1965.0215 (1+)	●	●	●	●	●	●	●	●	●	●	●	●	●	●	●	●	●		●	●
☑ 2041.0295 (1+)	●	●	●	●	●	●	●	●		●			●		●	●	●	●	●	
☑ 2078.9796 (1+)																				

☑ 2197.0497 (1+)																				
☑ 2237.0609 (1+)																				
☑ 2365.0120 (1+)																				
☑ 2409.9025 (1+)																				
☑ 2425.8507 (1+)	●	●	●	●	●	●	●		●	●	●		●	●	●	●	●		●	●
☑ 2433.0517 (1+)																				
☑ 2439.8554 (1+)																		●		
☑ 2442.8268 (1+)																				
☑ 2457.8373 (1+)																				
☑ 2471.8605 (1+)																				
☑ 2487.8369 (1+)																				
☑ 3052.6794 (1+)																				

Select All | Select None | Search Selected

Index

	Accession	Mass	Score	Description
1.	gi|294717812	80690	165	heat shock protein 90 [Triticum aestivum]
2.	gi|294717867	80724	165	heat shock protein 90 [Triticum dicoccoides]
3.	gi|326503698	80642	136	predicted protein [Hordeum vulgare subsp. vulgare]
4.	gi|294717810	80585	136	heat shock protein 90 [Triticum aestivum]
5.	gi|294717842	80626	136	heat shock protein 90 [Triticum urartu]
6.	gi|474225055	80657	136	Heat shock protein 81-3 [Triticum urartu]
7.	gi|294717814	80672	123	heat shock protein 90 [Triticum aestivum]
8.	gi|4204859	80619	123	heat shock protein 80 [Triticum aestivum]
9.	gi|743807690	80626	106	PREDICTED: heat shock protein 81-1-like [Elaeis guineensis]
10.	gi|357148345	80432	97	PREDICTED: heat shock protein 81-1 [Brachypodium distachyon]
11.	gi|743888516	65276	96	PREDICTED: heat shock protein 81-1, partial [Elaeis guineensis]
12.	gi|672181824	80164	91	PREDICTED: heat shock protein 81-1-like [Phoenix dactylifera]
13.	gi|799541145	68215	90	heat shock protein 90, partial [Siniperca chuatsi]
14.	gi|672193922	80435	90	PREDICTED: heat shock protein 81-1 [Phoenix dactylifera]
15.	gi|158513648	80429	89	RecName: Full=Heat shock protein 81-1; Short=HSP81-1; AltName: Full=Heat shock protein 82 [Oryza sativa Indica Group]
16.	gi|115477126	80023	88	Os08g0500700 [Oryza sativa Japonica Group]
17.	gi|573954616	80434	88	PREDICTED: heat shock protein 81-1-like isoform X2 [Oryza brachyantha]
18.	gi|720067771	80662	87	PREDICTED: heat shock protein 83-like [Nelumbo nucifera]
19.	gi|56202189	70944	87	putative heat shock protein 82 [Oryza sativa Japonica Group]
20.	gi|311303102	80562	87	heat shock protein 90 [Cenchrus americanus]

Results List

1. gi|294717812 **Mass:** 80690 **Score:** 165 **Expect:** 2.1e-09 **Matches:** 20
heat shock protein 90 [Triticum aestivum]

Observed	Mr(expt)	Mr(calc)	ppm	Start	End	Miss	Peptide
1081.4788	1080.4715	1080.5240	-48.57	320	328	0	R.APFDLFDTR.K
1127.5139	1126.5066	1126.5367	-26.67	420	429	0	K.LGVHEDSTNR.T
1201.6596	1200.6524	1200.6503	1.75	58	67	0	K.LDAQPELFIR.I
1237.6534	1236.6461	1236.6251	17.0	319	328	1	K.RAPFDLFDTR.K
1272.7172	1271.7099	1271.6834	20.9	89	100	0	K.SDLVNNLGTIAR.S
1320.7004	1319.6931	1319.6510	31.9	301	311	0	K.HFSVEGQLEFK.A
1416.8454	1415.8381	1415.7772	43.0	56	67	1	K.SKLDAQPELFIR.I
1527.8755	1526.8682	1526.7940	48.6	360	373	0	K.GIVDSEDLPLNISR.E
1541.8429	1540.8356	1540.7522	54.2	288	300	0	K.SLTNDWEEHLAVK.H
1544.9082	1543.9009	1543.8205	52.1	35	48	1	R.ELISNASDALDKIR.F
1583.8678	1582.8605	1582.7839	48.4	443	456	1	K.SGDELTSLKDYVTR.M
1609.9427	1608.9354	1608.8624	45.4	253	265	1	K.IKEVSHEWNLINK.Q
1789.9088	1788.9015	1788.8386	35.2	606	620	0	K.TMEINPENAIMEELR.K
1831.9592	1830.9519	1830.9039	26.2	273	287	1	R.KPEEITKDEYAAFYK.S
1836.9618	1835.9546	1835.9094	24.6	199	213	0	K.HSEFISYPISLWTEK.T
1917.9641	1916.9568	1916.9335	12.2	606	621	1	K.TMEINPENAIMEELRK.R
1933.9549	1932.9476	1932.9284	9.90	606	621	1	K.TMEINPENAIMEELRK.R + Oxidation (M)
1965.0215	1964.0142	1964.0043	5.04	198	213	1	K.KHSEFISYPISLWTEK.T
2041.0295	2040.0222	2040.0415	-9.46	175	190	1	K.ITLYLKDDQLEYLEER.R
2425.8507	2424.8434	2425.0571	-88.12	142	162	0	K.HNDDEQYVWESQAGGSFTVTR.D

No match to: 941.3557, 958.3910, 1222.7685, 1365.7740, 1384.8601, 1768.9015, 1869.9439, 2078.9796, 2197.0497, 2237.0609, 2365.0120, 2409.9025, 2433.0517, 2439.8554, 2442.8268, 2457.8373, 2471.8605, 2487.8369, 3052.6794

2. gi|294717867 **Mass:** 80724 **Score:** **165** **Expect:** 2.1e-09 **Matches:** 20

heat shock protein 90 [Triticum dicoccoides]

Observed	Mr(expt)	Mr(calc)	ppm	Start	End	Miss	Peptide
1081.4788	1080.4715	1080.5240	-48.57	320	328	0	R.APFDLFDTR.K
1127.5139	1126.5066	1126.5367	-26.67	420	429	0	K.LGVHEDSTNR.T
1201.6596	1200.6524	1200.6503	1.75	58	67	0	K.LDAQPELFIR.I
1237.6534	1236.6461	1236.6251	17.0	319	328	1	K.RAPFDLFDTR.K
1272.7172	1271.7099	1271.6834	20.9	89	100	0	K.SDLVNNLGTIAR.S
1320.7004	1319.6931	1319.6510	31.9	301	311	0	K.HFSVEGQLEFK.A
1416.8454	1415.8381	1415.7772	43.0	56	67	1	K.SKLDAQPELFIR.I
1527.8755	1526.8682	1526.7940	48.6	360	373	0	K.GIVDSEDLPLNISR.E
1541.8429	1540.8356	1540.7522	54.2	288	300	0	K.SLTNDWEEHLAVK.H
1544.9082	1543.9009	1543.8205	52.1	35	48	1	R.ELISNASDALDKIR.F
1583.8678	1582.8605	1582.7839	48.4	443	456	1	K.SGDELTSLKDYVTR.M
1609.9427	1608.9354	1608.8624	45.4	253	265	1	K.IKEVSHEWNLINK.Q
1789.9088	1788.9015	1788.8386	35.2	606	620	0	K.TMEINPENAIMEELR.K
1831.9592	1830.9519	1830.9039	26.2	273	287	1	R.KPEEITKDEYAAFYK.S
1836.9618	1835.9546	1835.9094	24.6	199	213	0	K.HSEFISYPISLWTEK.T
1917.9641	1916.9568	1916.9335	12.2	606	621	1	K.TMEINPENAIMEELRK.R
1933.9549	1932.9476	1932.9284	9.90	606	621	1	K.TMEINPENAIMEELRK.R + Oxidation (M)
1965.0215	1964.0142	1964.0043	5.04	198	213	1	K.KHSEFISYPISLWTEK.T
2041.0295	2040.0222	2040.0415	-9.46	175	190	1	K.ITLYLKDDQLEYLEER.R
2425.8507	2424.8434	2425.0571	-88.12	142	162	0	K.HNDDEQYVWESQAGGSFTVTR.D

No match to: 941.3557, 958.3910, 1222.7685, 1365.7740, 1384.8601, 1768.9015, 1869.9439, 2078.9796, 2197.0497, 2237.0609, 2365.0120, 2409.9025, 2433.0517, 2439.8554, 2442.8268, 2457.8373, 2471.8605, 2487.8369, 3052.6794

3. gi|326503698 **Mass:** 80642 **Score:** **136** **Expect:** 1.7e-06 **Matches:** 18

predicted protein [Hordeum vulgare subsp. vulgare]

Observed	Mr(expt)	Mr(calc)	ppm	Start	End	Miss	Peptide
1081.4788	1080.4715	1080.5240	-48.57	320	328	0	R.APFDLFDTR.K

1127.5139	1126.5066	1126.5367	-26.67	420	429	0	K.LGVHEDSTNR.T
1237.6534	1236.6461	1236.6251	17.0	319	328	1	K.RAPFDLFDTR.K
1272.7172	1271.7099	1271.6834	20.9	89	100	0	K.SDLVNNLGTIAR.S
1320.7004	1319.6931	1319.6510	31.9	301	311	0	K.HFSVEGQLEFK.A
1527.8755	1526.8682	1526.7940	48.6	360	373	0	K.GIVDSEDLPLNISR.E
1541.8429	1540.8356	1540.7522	54.2	288	300	0	K.SLTNDWEEHLAVK.H
1544.9082	1543.9009	1543.8205	52.1	35	48	1	R.ELISNASDALDKIR.F
1583.8678	1582.8605	1582.7839	48.4	443	456	1	K.SGDELTSLKDYVTR.M
1609.9427	1608.9354	1608.8624	45.4	253	265	1	K.IKEVSHEWNLINK.Q
1789.9088	1788.9015	1788.8386	35.2	606	620	0	K.TMEINPENAIMEELR.K
1831.9592	1830.9519	1830.9039	26.2	273	287	1	R.KPEEITKDEYAAFYK.S
1836.9618	1835.9546	1835.9094	24.6	199	213	0	K.HSEFISYPISLWTEK.T
1917.9641	1916.9568	1916.9335	12.2	606	621	1	K.TMEINPENAIMEELRK.R
1933.9549	1932.9476	1932.9284	9.90	606	621	1	K.TMEINPENAIMEELRK.R + Oxidation (M)
1965.0215	1964.0142	1964.0043	5.04	198	213	1	K.KHSEFISYPISLWTEK.T
2041.0295	2040.0222	2040.0415	-9.46	175	190	1	K.ITLYLKDDQLEYLEER.R
2425.8507	2424.8434	2425.0571	-88.12	142	162	0	K.HNDDEQYVWESQAGGSFTVTR.D

No match to: 941.3557, 958.3910, 1201.6596, 1222.7685, 1365.7740, 1384.8601, 1416.8454, 1768.9015, 1869.9439, 2078.9796, 2197.0497, 2237.0609, 2365.0120, 2409.9025, 2433.0517, 2439.8554, 2442.8268, 2457.8373, 2471.8605, 2487.8369, 3052.6794

4. gi|294717810 **Mass:** 80585 **Score:** **136** **Expect:** 1.7e-06 **Matches:** 18

heat shock protein 90 [Triticum aestivum]

Observed	Mr(expt)	Mr(calc)	ppm	Start	End	Miss	Peptide
1081.4788	1080.4715	1080.5240	-48.57	320	328	0	R.APFDLFDTR.K
1127.5139	1126.5066	1126.5367	-26.67	420	429	0	K.LGVHEDSTNR.T
1237.6534	1236.6461	1236.6251	17.0	319	328	1	K.RAPFDLFDTR.K
1272.7172	1271.7099	1271.6834	20.9	89	100	0	K.SDLVNNLGTIAR.S
1320.7004	1319.6931	1319.6510	31.9	301	311	0	K.HFSVEGQLEFK.A
1527.8755	1526.8682	1526.7940	48.6	360	373	0	K.GIVDSEDLPLNISR.E
1541.8429	1540.8356	1540.7522	54.2	288	300	0	K.SLTNDWEEHLAVK.H
1544.9082	1543.9009	1543.8205	52.1	35	48	1	R.ELISNASDALDKIR.F
1583.8678	1582.8605	1582.7839	48.4	443	456	1	K.SGDELTSLKDYVTR.M
1609.9427	1608.9354	1608.8624	45.4	253	265	1	K.IKEVSHEWNLINK.Q
1789.9088	1788.9015	1788.8386	35.2	606	620	0	K.TMEINPENAIMEELR.K
1831.9592	1830.9519	1830.9039	26.2	273	287	1	R.KPEEITKDEYAAFYK.S
1836.9618	1835.9546	1835.9094	24.6	199	213	0	K.HSEFISYPISLWTEK.T
1917.9641	1916.9568	1916.9335	12.2	606	621	1	K.TMEINPENAIMEELRK.R
1933.9549	1932.9476	1932.9284	9.90	606	621	1	K.TMEINPENAIMEELRK.R + Oxidation (M)
1965.0215	1964.0142	1964.0043	5.04	198	213	1	K.KHSEFISYPISLWTEK.T
2041.0295	2040.0222	2040.0415	-9.46	175	190	1	K.ITLYLKDDQLEYLEER.R
2425.8507	2424.8434	2425.0571	-88.12	142	162	0	K.HNDDEQYVWESQAGGSFTVTR.D

No match to: 941.3557, 958.3910, 1201.6596, 1222.7685, 1365.7740, 1384.8601, 1416.8454, 1768.9015, 1869.9439, 2078.9796, 2197.0497, 2237.0609, 2365.0120, 2409.9025, 2433.0517, 2439.8554, 2442.8268, 2457.8373, 2471.8605, 2487.8369, 3052.6794

5. gi|294717842 **Mass:** 80626 **Score:** **136** **Expect:** 1.7e-06 **Matches:** 18

heat shock protein 90 [Triticum urartu]

Observed	Mr(expt)	Mr(calc)	ppm	Start	End	Miss	Peptide
1081.4788	1080.4715	1080.5240	-48.57	320	328	0	R.APFDLFDTR.K
1127.5139	1126.5066	1126.5367	-26.67	420	429	0	K.LGVHEDSTNR.T
1237.6534	1236.6461	1236.6251	17.0	319	328	1	K.RAPFDLFDTR.K
1272.7172	1271.7099	1271.6834	20.9	89	100	0	K.SDLVNNLGTIAR.S
1320.7004	1319.6931	1319.6510	31.9	301	311	0	K.HFSVEGQLEFK.A
1527.8755	1526.8682	1526.7940	48.6	360	373	0	K.GIVDSEDLPLNISR.E
1541.8429	1540.8356	1540.7522	54.2	288	300	0	K.SLTNDWEEHLAVK.H

1544.9082	1543.9009	1543.8205	52.1	35 -	48	1	R.ELISNASDALDKIR.F
1583.8678	1582.8605	1582.7839	48.4	443 -	456	1	K.SGDELTSLKDYVTR.M
1609.9427	1608.9354	1608.8624	45.4	253 -	265	1	K.IKEVSHEWNLINK.Q
1789.9088	1788.9015	1788.8386	35.2	606 -	620	0	K.TMEINPENAIMEELR.K
1831.9592	1830.9519	1830.9039	26.2	273 -	287	1	R.KPEEITKDEYAAFYK.S
1836.9618	1835.9546	1835.9094	24.6	199 -	213	0	K.HSEFISYPISLWTEK.T
1917.9641	1916.9568	1916.9335	12.2	606 -	621	1	K.TMEINPENAIMEELRK.R
1933.9549	1932.9476	1932.9284	9.90	606 -	621	1	K.TMEINPENAIMEELRK.R + Oxidation (M)
1965.0215	1964.0142	1964.0043	5.04	198 -	213	1	K.KHSEFISYPISLWTEK.T
2041.0295	2040.0222	2040.0415	-9.46	175 -	190	1	K.ITLYLKDDQLEYLEER.R
2425.8507	2424.8434	2425.0571	-88.12	142 -	162	0	K.HNDDEQYVWESQAGGSFTVTR.D

No match to: 941.3557, 958.3910, 1201.6596, 1222.7685, 1365.7740, 1384.8601, 1416.8454, 1768.9015, 1869.9439, 2078.9796, 2197.0497, 2237.0609, 2365.0120, 2409.9025, 2433.0517, 2439.8554, 2442.8268, 2457.8373, 2471.8605, 2487.8369, 3052.6794

6. gi|474225055 **Mass:** 80657 **Score:** **136** **Expect:** 1.7e-06 **Matches:** 18

Heat shock protein 81-3 [Triticum urartu]

Observed	Mr(expt)	Mr(calc)	ppm	Start	End	Miss	Peptide
1081.4788	1080.4715	1080.5240	-48.57	320 -	328	0	R.APFDLFDTR.K
1127.5139	1126.5066	1126.5367	-26.67	420 -	429	0	K.LGVHEDSTNR.T
1237.6534	1236.6461	1236.6251	17.0	319 -	328	1	K.RAPFDLFDTR.K
1272.7172	1271.7099	1271.6834	20.9	89 -	100	0	K.SDLVNNLGTIAR.S
1320.7004	1319.6931	1319.6510	31.9	301 -	311	0	K.HFSVEGQLEFK.A
1527.8755	1526.8682	1526.7940	48.6	360 -	373	0	K.GIVDSEDLPLNISR.E
1541.8429	1540.8356	1540.7522	54.2	288 -	300	0	K.SLTNDWEEHLAVK.H
1544.9082	1543.9009	1543.8205	52.1	35 -	48	1	R.ELISNASDALDKIR.F
1583.8678	1582.8605	1582.7839	48.4	443 -	456	1	K.SGDELTSLKDYVTR.M
1609.9427	1608.9354	1608.8624	45.4	253 -	265	1	K.IKEVSHEWNLINK.Q
1789.9088	1788.9015	1788.8386	35.2	606 -	620	0	K.TMEINPENAIMEELR.K
1831.9592	1830.9519	1830.9039	26.2	273 -	287	1	R.KPEEITKDEYAAFYK.S
1836.9618	1835.9546	1835.9094	24.6	199 -	213	0	K.HSEFISYPISLWTEK.T
1917.9641	1916.9568	1916.9335	12.2	606 -	621	1	K.TMEINPENAIMEELRK.R
1933.9549	1932.9476	1932.9284	9.90	606 -	621	1	K.TMEINPENAIMEELRK.R + Oxidation (M)
1965.0215	1964.0142	1964.0043	5.04	198 -	213	1	K.KHSEFISYPISLWTEK.T
2041.0295	2040.0222	2040.0415	-9.46	175 -	190	1	K.ITLYLKDDQLEYLEER.R
2425.8507	2424.8434	2425.0571	-88.12	142 -	162	0	K.HNDDEQYVWESQAGGSFTVTR.D

No match to: 941.3557, 958.3910, 1201.6596, 1222.7685, 1365.7740, 1384.8601, 1416.8454, 1768.9015, 1869.9439, 2078.9796, 2197.0497, 2237.0609, 2365.0120, 2409.9025, 2433.0517, 2439.8554, 2442.8268, 2457.8373, 2471.8605, 2487.8369, 3052.6794

7. gi|294717814 **Mass:** 80672 **Score:** **123** **Expect:** 3.3e-05 **Matches:** 17

heat shock protein 90 [Triticum aestivum]

Observed	Mr(expt)	Mr(calc)	ppm	Start	End	Miss	Peptide
1081.4788	1080.4715	1080.5240	-48.57	320 -	328	0	R.APFDLFDTR.K
1127.5139	1126.5066	1126.5367	-26.67	420 -	429	0	K.LGVHEDSTNR.T
1237.6534	1236.6461	1236.6251	17.0	319 -	328	1	K.RAPFDLFDTR.K
1272.7172	1271.7099	1271.6834	20.9	89 -	100	0	K.SDLVNNLGTIAR.S
1320.7004	1319.6931	1319.6510	31.9	301 -	311	0	K.HFSVEGQLEFK.A
1527.8755	1526.8682	1526.7940	48.6	360 -	373	0	K.GIVDSEDLPLNISR.E
1541.8429	1540.8356	1540.7522	54.2	288 -	300	0	K.SLTNDWEEHLAVK.H
1544.9082	1543.9009	1543.8205	52.1	35 -	48	1	R.ELISNASDALDKIR.F
1583.8678	1582.8605	1582.7839	48.4	443 -	456	1	K.SGDELTSLKDYVTR.M
1609.9427	1608.9354	1608.8624	45.4	253 -	265	1	K.IKEVSHEWNLINK.Q
1789.9088	1788.9015	1788.8386	35.2	606 -	620	0	K.TMEINPENAIMEELR.K
1836.9618	1835.9546	1835.9094	24.6	199 -	213	0	K.HSEFISYPISLWTEK.T
1917.9641	1916.9568	1916.9335	12.2	606 -	621	1	K.TMEINPENAIMEELRK.R

1933.9549	1932.9476	1932.9284	9.90	606 -	621	1	K.TMEINPENAIMEELRK.R + Oxidation (M)
1965.0215	1964.0142	1964.0043	5.04	198 -	213	1	K.KHSEFISYPISLWTEK.T
2041.0295	2040.0222	2040.0415	-9.46	175 -	190	1	K.ITLYLKDDQLEYLEER.R
2425.8507	2424.8434	2425.0571	-88.12	142 -	162	0	K.HNDDEQYVWESQAGGSFTVTR.D

No match to: 941.3557, 958.3910, 1201.6596, 1222.7685, 1365.7740, 1384.8601, 1416.8454, 1768.9015, 1831.9592, 1869.9439, 2078.9796, 2197.0497, 2237.0609, 2365.0120, 2409.9025, 2433.0517, 2439.8554, 2442.8268, 2457.8373, 2471.8605, 2487.8369, 3052.6794

8. gi|4204859 **Mass:** 80619 **Score:** **123** **Expect:** 3.3e-05 **Matches:** 17

heat shock protein 80 [Triticum aestivum]

Observed	Mr(expt)	Mr(calc)	ppm	Start	End	Miss	Peptide
1081.4788	1080.4715	1080.5240	-48.57	320 -	328	0	R.APFDLFDTR.K
1127.5139	1126.5066	1126.5367	-26.67	420 -	429	0	K.LGVHEDSTNR.T
1201.6596	1200.6524	1200.6503	1.75	58 -	67	0	K.LDAQPELFIR.I
1237.6534	1236.6461	1236.6251	17.0	319 -	328	1	K.RAPFDLFDTR.K
1272.7172	1271.7099	1271.6834	20.9	89 -	100	0	K.SDLVNNLATIGR.S
1320.7004	1319.6931	1319.6510	31.9	301 -	311	0	K.HFSVEGQLEFK.A
1416.8454	1415.8381	1415.7772	43.0	56 -	67	1	K.SKLDAQPELFIR.I
1527.8755	1526.8682	1526.7940	48.6	360 -	373	0	K.GIVDSEDLPLNISR.E
1544.9082	1543.9009	1543.8205	52.1	35 -	48	1	R.ELISNASDALDKIR.F
1583.8678	1582.8605	1582.7839	48.4	443 -	456	1	K.SGDELTSLKDYVTR.M
1609.9427	1608.9354	1608.8624	45.4	253 -	265	1	K.IKEVSHEWNLINK.Q
1789.9088	1788.9015	1788.8386	35.2	606 -	620	0	K.TMEINPENAIMEELR.K
1836.9618	1835.9546	1835.9094	24.6	199 -	213	0	K.HSEFISYPISLWTEK.T
1917.9641	1916.9568	1916.9335	12.2	606 -	621	1	K.TMEINPENAIMEELRK.R
1933.9549	1932.9476	1932.9284	9.90	606 -	621	1	K.TMEINPENAIMEELRK.R + Oxidation (M)
1965.0215	1964.0142	1964.0043	5.04	198 -	213	1	K.KHSEFISYPISLWTEK.T
2041.0295	2040.0222	2040.0415	-9.46	175 -	190	1	K.ITLYLKDDQLEYLEER.R

No match to: 941.3557, 958.3910, 1222.7685, 1365.7740, 1384.8601, 1541.8429, 1768.9015, 1831.9592, 1869.9439, 2078.9796, 2197.0497, 2237.0609, 2365.0120, 2409.9025, 2425.8507, 2433.0517, 2439.8554, 2442.8268, 2457.8373, 2471.8605, 2487.8369, 3052.6794

9. gi|743807690 **Mass:** 80626 **Score:** **106** **Expect:** 0.0017 **Matches:** 14

PREDICTED: heat shock protein 81-1-like [Elaeis guineensis]

Observed	Mr(expt)	Mr(calc)	ppm	Start	End	Miss	Peptide
1081.4788	1080.4715	1080.5240	-48.57	322 -	330	0	R.APFDLFDTR.K
1237.6534	1236.6461	1236.6251	17.0	321 -	330	1	K.RAPFDLFDTR.K
1272.7172	1271.7099	1271.6834	20.9	89 -	100	0	K.SDLVNNLGTIAR.S
1320.7004	1319.6931	1319.6510	31.9	303 -	313	0	K.HFSVEGQLEFK.A
1527.8755	1526.8682	1526.7940	48.6	362 -	375	0	K.GIVDSEDLPLNISR.E
1541.8429	1540.8356	1540.7522	54.2	290 -	302	0	K.SLTNDWEEHLAVK.H
1544.9082	1543.9009	1543.8205	52.1	35 -	48	1	R.ELISNASDALDKIR.F
1583.8678	1582.8605	1582.7297	82.6	440 -	453	1	R.YHSTKSGDEMTSLK.D
1609.9427	1608.9354	1608.8029	82.4	73 -	88	0	K.GSNTLSIIDSGIGMTK.S + Oxidation (M)
1831.9592	1830.9519	1830.8491	56.2	608 -	622	0	K.TMEINPENPIMEELR.K + Oxidation (M)
1836.9618	1835.9546	1835.9094	24.6	199 -	213	0	K.HSEFISYPISLWTEK.T
1933.9549	1932.9476	1933.0156	-35.20	30 -	46	1	K.EIFLRELISNASDALDK.I
1965.0215	1964.0142	1964.0043	5.04	198 -	213	1	K.KHSEFISYPISLWTEK.T
2425.8507	2424.8434	2425.0571	-88.12	142 -	162	0	K.HNDDEQYVWESQAGGSFTVTR.D

No match to: 941.3557, 958.3910, 1127.5139, 1201.6596, 1222.7685, 1365.7740, 1384.8601, 1416.8454, 1768.9015, 1789.9088, 1869.9439, 1917.9641, 2041.0295, 2078.9796, 2197.0497, 2237.0609, 2365.0120, 2409.9025, 2433.0517, 2439.8554, 2442.8268, 2457.8373, 2471.8605, 2487.8369, 3052.6794

10. gi|357148345 **Mass:** 80432 **Score:** **97** **Expect:** 0.012 **Matches:** 15

PREDICTED: heat shock protein 81-1 [Brachypodium distachyon]

Observed	Mr(expt)	Mr(calc)	ppm	Start	End	Miss	Peptide
1081.4788	1080.4715	1080.5240	-48.57	319 -	327	0	R.APFDLFDTR.K

1127.5139	1126.5066	1126.5367	-26.67	419 - 428	0	K.LGVHEDSTNR.T
1237.6534	1236.6461	1236.6251	17.0	318 - 327	1	K.RAPFDLFDTR.K
1272.7172	1271.7099	1271.6834	20.9	89 - 100	0	K.SDLVNNLGTIAR.S
1320.7004	1319.6931	1319.6510	31.9	300 - 310	0	K.HFSVEGQLEFK.A
1527.8755	1526.8682	1526.7940	48.6	359 - 372	0	K.GIVDSEDLPLNISR.E
1541.8429	1540.8356	1540.7522	54.2	287 - 299	0	K.SLTNDWEEHLAVK.H
1583.8678	1582.8605	1582.7839	48.4	442 - 455	1	K.SGDELTSLKDYVTR.M
1789.9088	1788.9015	1788.8386	35.2	605 - 619	0	K.TMEINPENAIMEELR.K
1836.9618	1835.9546	1835.9094	24.6	198 - 212	0	K.HSEFISYPISLWTEK.T
1917.9641	1916.9568	1916.9335	12.2	605 - 620	1	K.TMEINPENAIMEELRK.R
1933.9549	1932.9476	1932.9284	9.90	605 - 620	1	K.TMEINPENAIMEELRK.R + Oxidation (M)
1965.0215	1964.0142	1964.0043	5.04	197 - 212	1	K.KHSEFISYPISLWTEK.T
2041.0295	2040.0222	2040.0415	-9.46	174 - 189	1	K.ITLYLKDDQLEYLEER.R
2425.8507	2424.8434	2425.0571	-88.12	142 - 162	0	K.HNDDEQYVWESQAGGSFTVTR.D

No match to: 941.3557, 958.3910, 1201.6596, 1222.7685, 1365.7740, 1384.8601, 1416.8454, 1544.9082, 1609.9427, 1768.9015, 1831.9592, 1869.9439, 2078.9796, 2197.0497, 2237.0609, 2365.0120, 2409.9025, 2433.0517, 2439.8554, 2442.8268, 2457.8373, 2471.8605, 2487.8369, 3052.6794

11. gi|743888516 **Mass:** 65276 **Score:** **96** **Expect:** 0.018 **Matches:** 12

PREDICTED: heat shock protein 81-1, partial [Elaeis guineensis]

Observed	Mr(expt)	Mr(calc)	ppm	Start - End	Miss	Peptide
1081.4788	1080.4715	1080.5240	-48.57	320 - 328	0	R.APFDLFDTR.K
1237.6534	1236.6461	1236.6251	17.0	319 - 328	1	K.RAPFDLFDTR.K
1272.7172	1271.7099	1271.6834	20.9	89 - 100	0	K.SDLVNNLGTIAR.S
1320.7004	1319.6931	1319.6510	31.9	301 - 311	0	K.HFSVEGQLEFK.A
1527.8755	1526.8682	1526.7940	48.6	360 - 373	0	K.GIVDSEDLPLNISR.E
1541.8429	1540.8356	1540.7522	54.2	288 - 300	0	K.SLTNDWEEHLAVK.H
1544.9082	1543.9009	1543.8205	52.1	35 - 48	1	R.ELISNASDALDKIR.F
1583.8678	1582.8605	1582.7297	82.6	438 - 451	1	R.YHSTKSGDEMTSLK.D
1836.9618	1835.9546	1835.9094	24.6	199 - 213	0	K.HSEFISYPISLWTEK.T
1933.9549	1932.9476	1933.0156	-35.20	30 - 46	1	K.EIFLRELISNASDALDK.I
1965.0215	1964.0142	1964.0043	5.04	198 - 213	1	K.KHSEFISYPISLWTEK.T
2425.8507	2424.8434	2425.0571	-88.12	142 - 162	0	K.HNDDEQYVWESQAGGSFTVTR.D

No match to: 941.3557, 958.3910, 1127.5139, 1201.6596, 1222.7685, 1365.7740, 1384.8601, 1416.8454, 1609.9427, 1768.9015, 1789.9088, 1831.9592, 1869.9439, 1917.9641, 2041.0295, 2078.9796, 2197.0497, 2237.0609, 2365.0120, 2409.9025, 2433.0517, 2439.8554, 2442.8268, 2457.8373, 2471.8605, 2487.8369, 3052.6794

12. gi|672181824 **Mass:** 80164 **Score:** **91** **Expect:** 0.05 **Matches:** 13

PREDICTED: heat shock protein 81-1-like [Phoenix dactylifera]

Observed	Mr(expt)	Mr(calc)	ppm	Start - End	Miss	Peptide
1081.4788	1080.4715	1080.5240	-48.57	320 - 328	0	R.APFDLFDTR.K
1237.6534	1236.6461	1236.6251	17.0	319 - 328	1	K.RAPFDLFDTR.K
1272.7172	1271.7099	1271.6834	20.9	89 - 100	0	K.SDLVNNLGTIAR.S
1320.7004	1319.6931	1319.6510	31.9	301 - 311	0	K.HFSVEGQLEFK.A
1527.8755	1526.8682	1526.7940	48.6	360 - 373	0	K.GIVDSEDLPLNISR.E
1541.8429	1540.8356	1540.7522	54.2	288 - 300	0	K.SLTNDWEEHLAVK.H
1544.9082	1543.9009	1543.8205	52.1	35 - 48	1	R.ELISNASDALDKIR.F
1583.8678	1582.8605	1582.7297	82.6	438 - 451	1	R.YHSTKSGDEMTSLK.D
1609.9427	1608.9354	1608.8029	82.4	73 - 88	0	K.ASNSLSIIDSGIGMTK.S + Oxidation (M)
1831.9592	1830.9519	1830.8491	56.2	606 - 620	0	K.TMEINPENPIMEELR.K + Oxidation (M)
1836.9618	1835.9546	1835.9094	24.6	199 - 213	0	K.HSEFISYPISLWTEK.T
1933.9549	1932.9476	1933.0156	-35.20	30 - 46	1	K.EIFLRELISNASDALDK.I
1965.0215	1964.0142	1964.0043	5.04	198 - 213	1	K.KHSEFISYPISLWTEK.T

No match to: 941.3557, 958.3910, 1127.5139, 1201.6596, 1222.7685, 1365.7740, 1384.8601, 1416.8454, 1768.9015, 1789.9088, 1869.9439, 1917.9641, 2041.0295, 2078.9796, 2197.0497, 2237.0609, 2409.9025, 2425.8507, 2433.0517, 2439.8554, 2442.8268, 2457.8373, 2471.8605, 2487.8369, 3052.6794

13. gi|799541145 **Mass:** 68215 **Score:** 90 **Expect:** 0.059 **Matches:** 12

heat shock protein 90, partial [Siniperca chuatsi]

Observed	Mr(expt)	Mr(calc)	ppm	Start	End	Miss	Peptide
1081.4788	1080.4715	1080.5240	-48.57	320	328	0	R.APFDLFDTR.K
1237.6534	1236.6461	1236.6251	17.0	319	328	1	K.RAPFDLFDTR.K
1272.7172	1271.7099	1271.6834	20.9	89	100	0	K.SDLVNNLGTIAR.S
1320.7004	1319.6931	1319.6510	31.9	301	311	0	K.HFSVEGQLEFK.A
1527.8755	1526.8682	1526.7940	48.6	360	373	0	K.GIVDSEDLPLNISR.E
1541.8429	1540.8356	1540.7522	54.2	288	300	0	K.SLTNDWEEHLAVK.H
1583.8678	1582.8605	1582.7839	48.4	443	456	1	K.SGDELTSLKDYVTR.M
1609.9427	1608.9354	1608.8029	82.4	73	88	0	K.ASNTLSIIDSGVGMTK.S + Oxidation (M)
1836.9618	1835.9546	1835.9094	24.6	199	213	0	K.HSEFISYPISLWTEK.T
1965.0215	1964.0142	1964.0043	5.04	198	213	1	K.KHSEFISYPISLWTEK.T
2041.0295	2040.0222	2040.0415	-9.46	175	190	1	K.ITLYLKDDQLEYLEER.R
2425.8507	2424.8434	2425.0571	-88.12	142	162	0	K.HNDDEQYVWESQAGGSFTVTR.D

No match to: 941.3557, 958.3910, 1127.5139, 1201.6596, 1222.7685, 1365.7740, 1384.8601, 1416.8454, 1544.9082, 1768.9015, 1789.9088, 1831.9592, 1869.9439, 1917.9641, 1933.9549, 2078.9796, 2197.0497, 2237.0609, 2365.0120, 2409.9025, 2433.0517, 2439.8554, 2442.8268, 2457.8373, 2471.8605, 2487.8369, 3052.6794

14. gi|672193922 **Mass:** 80435 **Score:** 90 **Expect:** 0.065 **Matches:** 13

PREDICTED: heat shock protein 81-1 [Phoenix dactylifera]

Observed	Mr(expt)	Mr(calc)	ppm	Start	End	Miss	Peptide
1081.4788	1080.4715	1080.5240	-48.57	320	328	0	R.APFDLFDTR.K
1237.6534	1236.6461	1236.6251	17.0	319	328	1	K.RAPFDLFDTR.K
1272.7172	1271.7099	1271.6834	20.9	89	100	0	K.SDLVNNLGTIAR.S
1320.7004	1319.6931	1319.6510	31.9	301	311	0	K.HFSVEGQLEFK.A
1527.8755	1526.8682	1526.7940	48.6	360	373	0	K.GIVDSEDLPLNISR.E
1541.8429	1540.8356	1540.7522	54.2	288	300	0	K.SLTNDWEEHLAVK.H
1544.9082	1543.9009	1543.8205	52.1	35	48	1	R.ELISNASDALDKIR.F
1583.8678	1582.8605	1582.7297	82.6	438	451	1	R.YHSTKSGDEMTSLK.D
1831.9592	1830.9519	1830.8491	56.2	606	620	0	K.TMEINPENPIMEELR.K + Oxidation (M)
1836.9618	1835.9546	1835.9094	24.6	199	213	0	K.HSEFISYPISLWTEK.T
1933.9549	1932.9476	1933.0156	-35.20	30	46	1	K.EIFLRELISNASDALDK.I
1965.0215	1964.0142	1964.0043	5.04	198	213	1	K.KHSEFISYPISLWTEK.T
2425.8507	2424.8434	2425.0571	-88.12	142	162	0	K.HNDDEQYVWESQAGGSFTVTR.D

No match to: 941.3557, 958.3910, 1127.5139, 1201.6596, 1222.7685, 1365.7740, 1384.8601, 1416.8454, 1609.9427, 1768.9015, 1789.9088, 1869.9439, 1917.9641, 2041.0295, 2078.9796, 2197.0497, 2237.0609, 2365.0120, 2409.9025, 2433.0517, 2439.8554, 2442.8268, 2457.8373, 2471.8605, 2487.8369, 3052.6794

15. gi|158513648 **Mass:** 80429 **Score:** 89 **Expect:** 0.088 **Matches:** 14

RecName: Full=Heat shock protein 81-1; Short=HSP81-1; AltName: Full=Heat shock protein 82 [Oryza sativa Indica Group]

Observed	Mr(expt)	Mr(calc)	ppm	Start	End	Miss	Peptide
1081.4788	1080.4715	1080.5240	-48.57	320	328	0	R.APFDLFDTR.K
1237.6534	1236.6461	1236.6251	17.0	319	328	1	K.RAPFDLFDTR.K
1272.7172	1271.7099	1271.6834	20.9	89	100	0	K.SDLVNNLGTIAR.S
1320.7004	1319.6931	1319.6510	31.9	301	311	0	K.HFSVEGQLEFK.A
1527.8755	1526.8682	1526.7940	48.6	360	373	0	K.GIVDSEDLPLNISR.E
1541.8429	1540.8356	1540.7522	54.2	288	300	0	K.SLTNDWEEHLAVK.H
1583.8678	1582.8605	1582.7839	48.4	443	456	1	K.SGDELTSLKDYVTR.M
1789.9088	1788.9015	1788.8386	35.2	606	620	0	K.TMEINPENAIMEELR.K
1836.9618	1835.9546	1835.9094	24.6	199	213	0	K.HSEFISYPISLWTEK.T
1917.9641	1916.9568	1916.9335	12.2	606	621	1	K.TMEINPENAIMEELRK.R
1933.9549	1932.9476	1932.9284	9.90	606	621	1	K.TMEINPENAIMEELRK.R + Oxidation (M)
1965.0215	1964.0142	1964.0043	5.04	198	213	1	K.KHSEFISYPISLWTEK.T
2041.0295	2040.0222	2040.0415	-9.46	175	190	1	K.ITLYLKDDQLEYLEER.R
2425.8507	2424.8434	2425.0571	-88.12	142	162	0	K.HNDDEQYVWESQAGGSFTVTR.D

No match to: 941.3557, 958.3910, 1127.5139, 1201.6596, 1222.7685, 1365.7740, 1384.8601, 1416.8454, 1544.9082, 1609.9427, 1768.9015, 1831.9592, 1869.9439, 2078.9796, 2197.0497, 2237.0609, 2365.0120, 2409.9025, 2433.0517, 2439.8554, 2442.8268, 2457.8373, 2471.8605, 2487.8369, 3052.6794

16. gi|115477126 **Mass:** 80023 **Score:** 88 **Expect:** 0.098 **Matches:** 14

Os08g0500700 [Oryza sativa Japonica Group]

Observed	Mr(expt)	Mr(calc)	ppm	Start	End	Miss	Peptide
1081.4788	1080.4715	1080.5240	-48.57	315	323	0	R.APFDLFDTR.K
1237.6534	1236.6461	1236.6251	17.0	314	323	1	K.RAPFDLFDTR.K
1272.7172	1271.7099	1271.6834	20.9	84	95	0	K.SDLVNNLGTIAR.S
1320.7004	1319.6931	1319.6510	31.9	296	306	0	K.HFSVEGQLEFK.A
1527.8755	1526.8682	1526.7940	48.6	355	368	0	K.GIVDSEDLPLNISR.E
1541.8429	1540.8356	1540.7522	54.2	283	295	0	K.SLTNDWEEHLAVK.H
1583.8678	1582.8605	1582.7839	48.4	438	451	1	K.SGDELTSLKDYVTR.M
1789.9088	1788.9015	1788.8386	35.2	601	615	0	K.TMEINPENAIMEELR.K
1836.9618	1835.9546	1835.9094	24.6	194	208	0	K.HSEFISYPISLWTEK.T
1917.9641	1916.9568	1916.9335	12.2	601	616	1	K.TMEINPENAIMEELRK.R
1933.9549	1932.9476	1932.9284	9.90	601	616	1	K.TMEINPENAIMEELRK.R + Oxidation (M)
1965.0215	1964.0142	1964.0043	5.04	193	208	1	K.KHSEFISYPISLWTEK.T
2041.0295	2040.0222	2040.0415	-9.46	170	185	1	K.ITLYLKDDQLEYLEER.R
2425.8507	2424.8434	2425.0571	-88.12	137	157	0	K.HNDDEQYVWESQAGGSFTVTR.D

No match to: 941.3557, 958.3910, 1127.5139, 1201.6596, 1222.7685, 1365.7740, 1384.8601, 1416.8454, 1544.9082, 1609.9427, 1768.9015, 1831.9592, 1869.9439, 2078.9796, 2197.0497, 2237.0609, 2365.0120, 2409.9025, 2433.0517, 2439.8554, 2442.8268, 2457.8373, 2471.8605, 2487.8369, 3052.6794

17. gi|573954616 **Mass:** 80434 **Score:** 88 **Expect:** 0.11 **Matches:** 14

PREDICTED: heat shock protein 81-1-like isoform X2 [Oryza brachyantha]

Observed	Mr(expt)	Mr(calc)	ppm	Start	End	Miss	Peptide
1081.4788	1080.4715	1080.5240	-48.57	320	328	0	R.APFDLFDTR.K
1237.6534	1236.6461	1236.6251	17.0	319	328	1	K.RAPFDLFDTR.K
1272.7172	1271.7099	1271.6834	20.9	89	100	0	K.SDLVNNLGTIAR.S
1320.7004	1319.6931	1319.6510	31.9	301	311	0	K.HFSVEGQLEFK.A
1527.8755	1526.8682	1526.7940	48.6	360	373	0	K.GIVDSEDLPLNISR.E
1541.8429	1540.8356	1540.7522	54.2	288	300	0	K.SLTNDWEEHLAVK.H
1583.8678	1582.8605	1582.7839	48.4	443	456	1	K.SGDELTSLKDYVTR.M
1789.9088	1788.9015	1788.8386	35.2	606	620	0	K.TMEINPENAIMEELR.K
1836.9618	1835.9546	1835.9094	24.6	199	213	0	K.HSEFISYPISLWTEK.T
1917.9641	1916.9568	1916.9335	12.2	606	621	1	K.TMEINPENAIMEELRK.R
1933.9549	1932.9476	1932.9284	9.90	606	621	1	K.TMEINPENAIMEELRK.R + Oxidation (M)
1965.0215	1964.0142	1964.0043	5.04	198	213	1	K.KHSEFISYPISLWTEK.T
2041.0295	2040.0222	2040.0415	-9.46	175	190	1	K.ITLYLKDDQLEYLEER.R
2425.8507	2424.8434	2425.0571	-88.12	142	162	0	K.HNDDEQYVWESQAGGSFTVTR.D

No match to: 941.3557, 958.3910, 1127.5139, 1201.6596, 1222.7685, 1365.7740, 1384.8601, 1416.8454, 1544.9082, 1609.9427, 1768.9015, 1831.9592, 1869.9439, 2078.9796, 2197.0497, 2237.0609, 2365.0120, 2409.9025, 2433.0517, 2439.8554, 2442.8268, 2457.8373, 2471.8605, 2487.8369, 3052.6794

18. gi|720067771 **Mass:** 80662 **Score:** 87 **Expect:** 0.12 **Matches:** 13

PREDICTED: heat shock protein 83-like [Nelumbo nucifera]

Observed	Mr(expt)	Mr(calc)	ppm	Start	End	Miss	Peptide
1081.4788	1080.4715	1080.5240	-48.57	321	329	0	R.APFDLFDTR.K
1201.6596	1200.6524	1200.6503	1.75	62	71	0	K.LDAQPELFIR.L
1237.6534	1236.6461	1236.6251	17.0	320	329	1	K.RAPFDLFDTR.K
1320.7004	1319.6931	1319.6510	31.9	302	312	0	K.HFSVEGQLEFK.A
1416.8454	1415.8381	1415.7772	43.0	60	71	1	K.SKLDAQPELFIR.L
1527.8755	1526.8682	1526.7365	86.3	289	301	0	K.SLTNDWEDHLAVK.H
1544.9082	1543.9009	1543.8205	52.1	39	52	1	R.ELISNASDALDKIR.F
1583.8678	1582.8605	1582.7839	48.4	444	457	1	K.SGDELTSLKDYVTR.M

Observed	Mr(expt)	Mr(calc)	ppm	Start	End	Miss	Peptide
1609.9427	1608.9354	1608.8624	45.4	254 -	266	1	K.VKEVSHEWQLINK.Q
1768.9015	1767.8942	1767.7534	79.7	218 -	232	1	K.TTEKEISDDEDEETK.K
1933.9549	1932.9476	1933.0156	-35.20	34 -	50	1	K.EIFLRELISNASDALDK.I
2041.0295	2040.0222	2040.0356	-6.59	202 -	217	1	K.KHSEFISYPIYLWTEK.T
2439.8554	2438.8482	2439.0727	-92.07	146 -	166	0	K.HNDDEQYIWESQAGGSFTVTR.D

No match to: 941.3557, 958.3910, 1127.5139, 1222.7685, 1272.7172, 1365.7740, 1384.8601, 1541.8429, 1789.9088, 1831.9592, 1836.9618, 1869.9439, 1917.9641, 1965.0215, 2078.9796, 2197.0497, 2237.0609, 2365.0120, 2409.9025, 2425.8507, 2433.0517, 2442.8268, 2457.8373, 2471.8605, 2487.8369, 3052.6794

19. gi|56202189 **Mass:** 70944 **Score:** 87 **Expect:** 0.14 **Matches:** 14

putative heat shock protein 82 [Oryza sativa Japonica Group]

Observed	Mr(expt)	Mr(calc)	ppm	Start	End	Miss	Peptide
1081.4788	1080.4715	1080.5240	-48.57	235 -	243	0	R.APFDLFDTR.K
1237.6534	1236.6461	1236.6251	17.0	234 -	243	1	K.RAPFDLFDTR.K
1272.7172	1271.7099	1271.6834	20.9	4 -	15	0	K.SDLVNNLGTIAR.S
1320.7004	1319.6931	1319.6510	31.9	216 -	226	0	K.HFSVEGQLEFK.A
1527.8755	1526.8682	1526.7940	48.6	275 -	288	0	K.GIVDSEDLPLNISR.E
1541.8429	1540.8356	1540.7522	54.2	203 -	215	0	K.SLTNDWEEHLAVK.H
1583.8678	1582.8605	1582.7839	48.4	358 -	371	1	K.SGDELTSLKDYVTR.M
1789.9088	1788.9015	1788.8386	35.2	521 -	535	0	K.TMEINPENAIMEELR.K
1836.9618	1835.9546	1835.9094	24.6	114 -	128	0	K.HSEFISYPISLWTEK.T
1917.9641	1916.9568	1916.9335	12.2	521 -	536	1	K.TMEINPENAIMEELRK.R
1933.9549	1932.9476	1932.9284	9.90	521 -	536	1	K.TMEINPENAIMEELRK.R + Oxidation (M)
1965.0215	1964.0142	1964.0043	5.04	113 -	128	1	K.KHSEFISYPISLWTEK.T
2041.0295	2040.0222	2040.0415	-9.46	90 -	105	1	K.ITLYLKDDQLEYLEER.R
2425.8507	2424.8434	2425.0571	-88.12	57 -	77	0	K.HNDDEQYVWESQAGGSFTVTR.D

No match to: 941.3557, 958.3910, 1127.5139, 1201.6596, 1222.7685, 1365.7740, 1384.8601, 1416.8454, 1544.9082, 1609.9427, 1768.9015, 1831.9592, 1869.9439, 2078.9796, 2197.0497, 2237.0609, 2365.0120, 2409.9025, 2433.0517, 2439.8554, 2442.8268, 2457.8373, 2471.8605, 2487.8369, 3052.6794

20. gi|311303102 **Mass:** 80562 **Score:** 87 **Expect:** 0.14 **Matches:** 14

heat shock protein 90 [Cenchrus americanus]

Observed	Mr(expt)	Mr(calc)	ppm	Start	End	Miss	Peptide
1081.4788	1080.4715	1080.5240	-48.57	319 -	327	0	R.APFDLFDTR.K
1237.6534	1236.6461	1236.6251	17.0	318 -	327	1	K.RAPFDLFDTR.K
1272.7172	1271.7099	1271.6834	20.9	89 -	100	0	K.SDLVNNLGTIAR.S
1320.7004	1319.6931	1319.6510	31.9	300 -	310	0	K.HFSVEGQLEFK.A
1527.8755	1526.8682	1526.7940	48.6	359 -	372	0	K.GIVDSEDLPLNISR.E
1541.8429	1540.8356	1540.7522	54.2	287 -	299	0	K.SLTNDWEEHLAVK.H
1583.8678	1582.8605	1582.7839	48.4	442 -	455	1	K.SGDELTSLKDYVTR.M
1609.9427	1608.9354	1608.8624	45.4	252 -	264	1	K.IKEVSHEWQLVNK.Q
1789.9088	1788.9015	1788.8386	35.2	605 -	619	0	K.TMEINPENAIMEELR.K
1836.9618	1835.9546	1835.9094	24.6	199 -	213	0	K.HSEFISYPISLWTEK.T
1917.9641	1916.9568	1916.9335	12.2	605 -	620	1	K.TMEINPENAIMEELRK.R
1933.9549	1932.9476	1932.9284	9.90	605 -	620	1	K.TMEINPENAIMEELRK.R + Oxidation (M)
1965.0215	1964.0142	1964.0043	5.04	198 -	213	1	K.KHSEFISYPISLWTEK.T
2425.8507	2424.8434	2425.0571	-88.12	142 -	162	0	K.HNDDEQYVWESQAGGSFTVTR.D

No match to: 941.3557, 958.3910, 1127.5139, 1201.6596, 1222.7685, 1365.7740, 1384.8601, 1416.8454, 1544.9082, 1768.9015, 1831.9592, 1869.9439, 2041.0295, 2078.9796, 2197.0497, 2237.0609, 2365.0120, 2409.9025, 2433.0517, 2439.8554, 2442.8268, 2457.8373, 2471.8605, 2487.8369, 3052.6794

Search Parameters

Type of search : **Peptide Mass Fingerprint**
Enzyme : **Trypsin**
Fixed modifications : **Carbamidomethyl (C)**

```
Variable modifications : Oxidation (M)
Mass values            : Monoisotopic
Protein Mass           : Unrestricted
Peptide Mass Tolerance : ± 100 ppm
Peptide Charge State   : 1+
Max Missed Cleavages   : 1
Number of queries      : 39
```

Mascot: http://www.matrixscience.com/
