# Supplementary material for: Heat stress transcripts, differential expression, and profiling of heat stress tolerant gene TaHsp90 in Indian wheat (Triticum aestivum L.) cv C306
Source: PLoS One. 2018 Jun 25;13(6):e0198293. doi: 10.1371/journal.pone.0198293 (PMC6016904; doi:10.1371/journal.pone.0198293)
Supplement: S1 Table — (DOCX) [file pone.0198293.s010.docx]

Table .. Enriched biological processes related GO terms under HS at 37°C

| GO term | Ontology | Description | *p*-value | FDR |
| --- | --- | --- | --- | --- |
| GO:0006457 | P | protein folding | 0.0000021 | 0.0000021 |
| GO:0044267 | P | cellular protein metabolic process | 0.000045 | 0.000045 |
| GO:0019538 | P | protein metabolic process | 0.000076 | 0.000076 |
| GO:0044237 | P | cellular metabolic process | 0.00057 | 0.00057 |
| GO:0008152 | P | metabolic process | 0.0019 | 0.0019 |
| GO:0015672 | P | monovalent inorganic cation transport | 0.0046 | 0.0046 |
| GO:0043170 | P | macromolecule metabolic process | 0.0094 | 0.0094 |
| GO:0009987 | P | cellular process | 0.015 | 0.015 |
| GO:0044248 | P | cellular catabolic process | 0.016 | 0.016 |
| GO:0044260 | P | cellular macromolecule metabolic process | 0.018 | 0.018 |
| GO:0006950 | P | response to stress | 0.019 | 0.019 |
| GO:0006812 | P | cation transport | 0.023 | 0.023 |
| GO:0044238 | P | primary metabolic process | 0.03 | 0.03 |
| GO:0006412 | P | translation | 0.034 | 0.034 |
| GO:0006091 | P | generation of precursor metabolites and energy | 0.046 | 0.046 |
| GO:0051082 | F | unfolded protein binding | 0.000000094 | 0.000000094 |
| GO:0070011 | F | peptidase activity, acting on L-amino acid peptides | 0.032 | 0.032 |
| GO:0009579 | C | thylakoid | 0.0066 | 0.0066 |
| GO:0044424 | C | intracellular part | 0.012 | 0.012 |
| GO:0005622 | C | intracellular | 0.015 | 0.015 |
| GO:0031090 | C | organelle membrane | 0.03 | 0.03 |
| GO:0043229 | C | intracellular organelle | 0.038 | 0.038 |
| GO:0043226 | C | organelle | 0.038 | 0.038 |
| GO:0009507 | C | chloroplast | 0.04 | 0.04 |
| GO:0030529 | C | ribonucleoprotein complex | 0.041 | 0.041 |

Table .. Enriched biological processes related GO terms under HS at 42°C

| **GO term** | **Ontology** | **Description** | ***p*-value** | **FDR** |
| --- | --- | --- | --- | --- |
| GO:0006457 | P | protein folding | 0.000025 | 0.000025 |
| GO:0019538 | P | protein metabolic process | 0.00012 | 0.00012 |
| GO:0044267 | P | cellular protein metabolic process | 0.00018 | 0.00018 |
| GO:0043581 | P | mycelium development | 0.00075 | 0.00075 |
| GO:0044237 | P | cellular metabolic process | 0.002 | 0.002 |
| GO:0008152 | P | metabolic process | 0.0035 | 0.0035 |
| GO:0044260 | P | cellular macromolecule metabolic process | 0.0044 | 0.0044 |
| GO:0043170 | P | macromolecule metabolic process | 0.0044 | 0.0044 |
| GO:0006412 | P | translation | 0.0051 | 0.0051 |
| GO:0042254 | P | ribosome biogenesis | 0.0055 | 0.0055 |
| GO:0009987 | P | cellular process | 0.0058 | 0.0058 |
| GO:0022613 | P | ribonucleoprotein complex biogenesis | 0.006 | 0.006 |
| GO:0044238 | P | primary metabolic process | 0.011 | 0.011 |
| GO:0044085 | P | cellular component biogenesis | 0.02 | 0.02 |
| GO:0006950 | P | response to stress | 0.023 | 0.023 |
| GO:0010467 | P | gene expression | 0.036 | 0.036 |
| GO:0032501 | P | multicellular organismal process | 0.068 | 0.068 |
| GO:0034641 | P | cellular nitrogen compound metabolic process | 0.07 | 0.07 |
| GO:0050896 | P | response to stimulus | 0.075 | 0.075 |
| GO:0048856 | P | anatomical structure development | 0.075 | 0.075 |
| GO:0003735 | F | structural constituent of ribosome | 0.0014 | 0.0014 |
| GO:0005198 | F | structural molecule activity | 0.0018 | 0.0018 |
| GO:0030554 | F | adenyl nucleotide binding | 0.0091 | 0.0091 |
| GO:0001883 | F | purine nucleoside binding | 0.0091 | 0.0091 |
| GO:0001882 | F | nucleoside binding | 0.0091 | 0.0091 |
| GO:0005488 | F | binding | 0.015 | 0.015 |
| GO:0000166 | F | nucleotide binding | 0.021 | 0.021 |
| GO:0003723 | F | RNA binding | 0.024 | 0.024 |
| GO:0005524 | F | ATP binding | 0.025 | 0.025 |
| GO:0032559 | F | adenyl ribonucleotide binding | 0.026 | 0.026 |
| GO:0017076 | F | purine nucleotide binding | 0.03 | 0.03 |
| GO:0008270 | F | zinc ion binding | 0.038 | 0.038 |
| GO:0046872 | F | metal ion binding | 0.042 | 0.042 |
| GO:0016491 | F | oxidoreductase activity | 0.06 | 0.06 |
| GO:0043169 | F | cation binding | 0.063 | 0.063 |
| GO:0043167 | F | ion binding | 0.064 | 0.064 |
| GO:0032555 | F | purine ribonucleotide binding | 0.073 | 0.073 |
| GO:0032553 | F | ribonucleotide binding | 0.073 | 0.073 |
| GO:0015935 | C | small ribosomal subunit | 0.00029 | 0.00029 |
| GO:0033279 | C | ribosomal subunit | 0.0037 | 0.0037 |
| GO:0043232 | C | intracellular non-membrane-bounded organelle | 0.0038 | 0.0038 |
| GO:0043228 | C | non-membrane-bounded organelle | 0.0038 | 0.0038 |
| GO:0005840 | C | ribosome | 0.0064 | 0.0064 |
| GO:0030529 | C | ribonucleoprotein complex | 0.0067 | 0.0067 |
| GO:0016020 | C | membrane | 0.035 | 0.035 |
| GO:0044446 | C | intracellular organelle part | 0.045 | 0.045 |
| GO:0044422 | C | organelle part | 0.046 | 0.046 |
| GO:0032991 | C | macromolecular complex | 0.061 | 0.061 |
| GO:0005886 | C | plasma membrane | 0.075 | 0.075 |
